# Complement activation by IgG subclasses is governed by their ability to oligomerize upon antigen binding

**DOI:** 10.1073/pnas.2406192121

**Published:** 2024-10-22

**Authors:** Nikolaus Frischauf, Jürgen Strasser, Ellen G.F. Borg, Aran F. Labrijn, Frank J. Beurskens, Johannes Preiner

**Affiliations:** ^a^Medical Engineering, Nano Structuring and Bio-Analytics, University of Applied Sciences Upper Austria, Linz 4020, Austria; ^b^Genmab, Utrecht 3584 CT, The Netherlands

**Keywords:** classical complement pathway, C1, IgG subclasses, IgG oligomerization, Fc–Fc interactions

## Abstract

Complement activation through antibody–antigen complexes is crucial in various pathophysiological processes and utilized in immunotherapies to eliminate infectious agents, regulatory immune cells, or cancer cells. The tertiary structures of the four IgG antibody subclasses are largely comparable, with the most prominent difference being the hinge regions connecting the Fab and Fc domains, providing them with unique structural flexibility. Complement recruitment and activation depend strongly on IgG subclass, which is commonly rationalized by differences in hinge flexibility and the respective affinities for C1, the first component of the classical complement pathway. However, a unifying mechanism of how these different IgG subclass properties combine to modulate C1 activation has not yet been proposed. We here demonstrate that complement activation is determined by their varying ability to form IgG oligomers on antigenic surfaces large enough to multivalently bind and activate C1. We directly visualize the resulting IgG oligomer structures and characterize their distribution by means of high-speed atomic force microscopy, quantify their complement recruitment efficiency from quartz crystal microbalance experiments, and characterize their ability to activate complement on tumor cell lines as well as in vesicle-based complement lysis assays. We present a mechanistic model of the multivalent interactions that govern C1 binding to IgG oligomers and use it to extract kinetic rate constants from real-time interaction data from which we further calculate equilibrium dissociation constants. Together, we provide a comprehensive view on the parameters that govern complement activation by the different IgG subclasses, which may inform the design of future antibody therapies.

## Significance

The classical complement pathway is an important effector mechanism for clearance of infections, which is also employed in immunotherapies to eliminate tumor cells. Its initiation is governed by the binding and activation of C1 to multiple IgG antibody Fc domains. The ability to activate complement differs strongly between the four IgG subclasses; however, a unifying mechanism rationalizing the respective potencies to activate complement has not been established. We here show that complement activation is determined by the respective ability of IgG1-4 to form large oligomers on antigenic surfaces that can multivalently bind C1. We provide a comprehensive mechanistic framework of this process and reveal how C1 binding to large IgG oligomers is linked to target cell clearance.

Immunoglobulin G (IgG) is the most abundant antibody isotype in human serum and a highly potent effector molecule. IgG is divided into four subclasses designated IgG1, IgG2, IgG3, and IgG4 in order of decreasing abundance. Clearance of IgG-opsonized pathogens or cells is facilitated by various effector mechanisms of the innate immune system, including the classical complement pathway, an amplifiable cascade of soluble zymogens abundant in blood and other extracellular fluids. It is initiated by multivalent binding of zymogen C1, consisting of the hexavalent recognition protein C1q (made of six collagen arms assembled in a “bunch of tulips” like structure, each ending in a globular IgG-Fc binding domain; gC1q) and a heterotetramer of serin proteases C1r and C1s (C1qr_2_s_2_), to antibody–antigen complexes (1–3). Rearrangements within C1 as a consequence of antibody recognition by C1q activate the proteases that are then able to cleave complement proteins C4 and C2 into C4a,b and C2a,b, with the respective b products potentially being covalently deposited on the antigenic membrane. In this way, the formation of downstream enzymatic complexes, the C3 and C5 convertases (C4b2b and C4b2b3b) is induced, which then may lead to the formation of the membrane attack complex (MAC, C5b-9), a lytic pore, that is inserted into the target cell membrane.

Although the four IgG subclasses are more than 90% identical on the amino acid level (4), with the most prominent difference being the hinge region (length of 15 aa, 12 aa, 62aa, and 12 aa in IgG1-4, respectively) connecting the Fc domain with the two Fab domains, complement recruitment and further activation depend strongly on subclass. These functional differences have been associated with the respective monovalent affinity for C1q (4), decreasing in the order IgG3 > IgG1 > IgG2 > IgG4 (5), and with the hinge-mediated relative flexibility of the Fab arms with respect to the Fc, found to be in the order IgG3 > IgG1 > IgG4 > IgG2 (6, 7). Given their low affinity for C1q, with equilibrium dissociation constants of the monovalent interactions ranging between 34 µM (IgG3) and 229 µM (IgG4) (4), it was also established that significant C1 binding at physiological concentrations [~0.17 µM (8)] requires multivalent interactions between C1 and multiple IgGs (9–16). However, a unified framework of how these factors are interlinked and govern the actual ability/inability of a certain IgG subclass to activate complement on antigenic surfaces has not yet been proposed.

We have recently shown that antigen-mediated oligomerization of IgG1 via noncovalent Fc–Fc interactions is crucial for engaging the complement system through C1(q) binding. While antigen-bound IgG1 may exist as a distribution of oligomers ranging from monomers to hexamers, only tetramers, pentamers, and hexamers were capable of inducing complement-dependent cytotoxicity (CDC), as a minimum of four gC1q headpieces was required to bind in close proximity to adjacent subunits within IgG1 oligomers (17). Accordingly, tetravalent C1 binding to nonadjacent subunits within an IgG1 hexamer did not induce CDC, underlining the necessity for a particular binding geometry and valency that results in sufficient compaction of C1q arms to induce conformational rearrangements that allow C1r to activate C1s (2).

The existence of IgG hexamers on antigenic surfaces has been experimentally proven only for the IgG1 and IgG3 subclasses (1, 2, 18), while the dynamic formation of differently sized IgG oligomers on antigenic surfaces and the impact of the CDC-enhancing E430G point mutation on these distributions have solely been demonstrated for the IgG1 subclass (17, 19). Introduction of this mutation into the four subclass variants of an anti-WTA IgG enhanced C3b deposition on bacteria (20), and another point mutation (E345R) that induces up to 1% IgG1-E345R hexamers in solution (21) was found to increase CDC of lymphoma cells by all four subclasses (1). It thus seems likely that all IgG subclasses need to assemble into similar oligomeric structures that result in a common Fc-platform allowing for at least tetravalent C1 binding to adjacent IgG monomers within the oligomer to activate complement.

In this study, we demonstrate that antigen-dependent IgG oligomerization is a general mechanism inherent to all IgG subclasses and the main prerequisite for complement C1 binding and activation. We performed CDC assays to systematically assess and compare the efficacy of IgG subclasses as well as the effect of the IgG1-oligomerization enhancing E430G point mutation on all IgG subclasses on killing different lymphoma cell lines. Using high-speed atomic force microscopy (HS-AFM), we visualize the respective oligomer structures formed on antigenic supported lipid bilayers and characterize the oligomerization propensity of IgG subclasses as well as the E430G point mutants thereof. We further study complement C1 and C1q binding to IgG1-4 oligomers on antigenic membranes in quartz crystal microbalance (QCM) experiments to determine kinetic rate constants, equilibrium dissociation constants, and resulting complement recruitment efficiencies at various antigen densities. Finally, we perform liposomal vesicle-based complement lysis assays to link our single molecule observations to terminal complement activation on antigen-coated vesicles. We highlight a complex interplay of IgG flexibility, multivalent IgG Fc–Fc, and IgG–C1(q) interactions, that determine the IgG subclass–specific differences in complement recruitment and activation.

## Results

### CDC Efficacy Depends on IgG Subclass and can be Enhanced by the E430G Point Mutation.

**Fig. 1.**
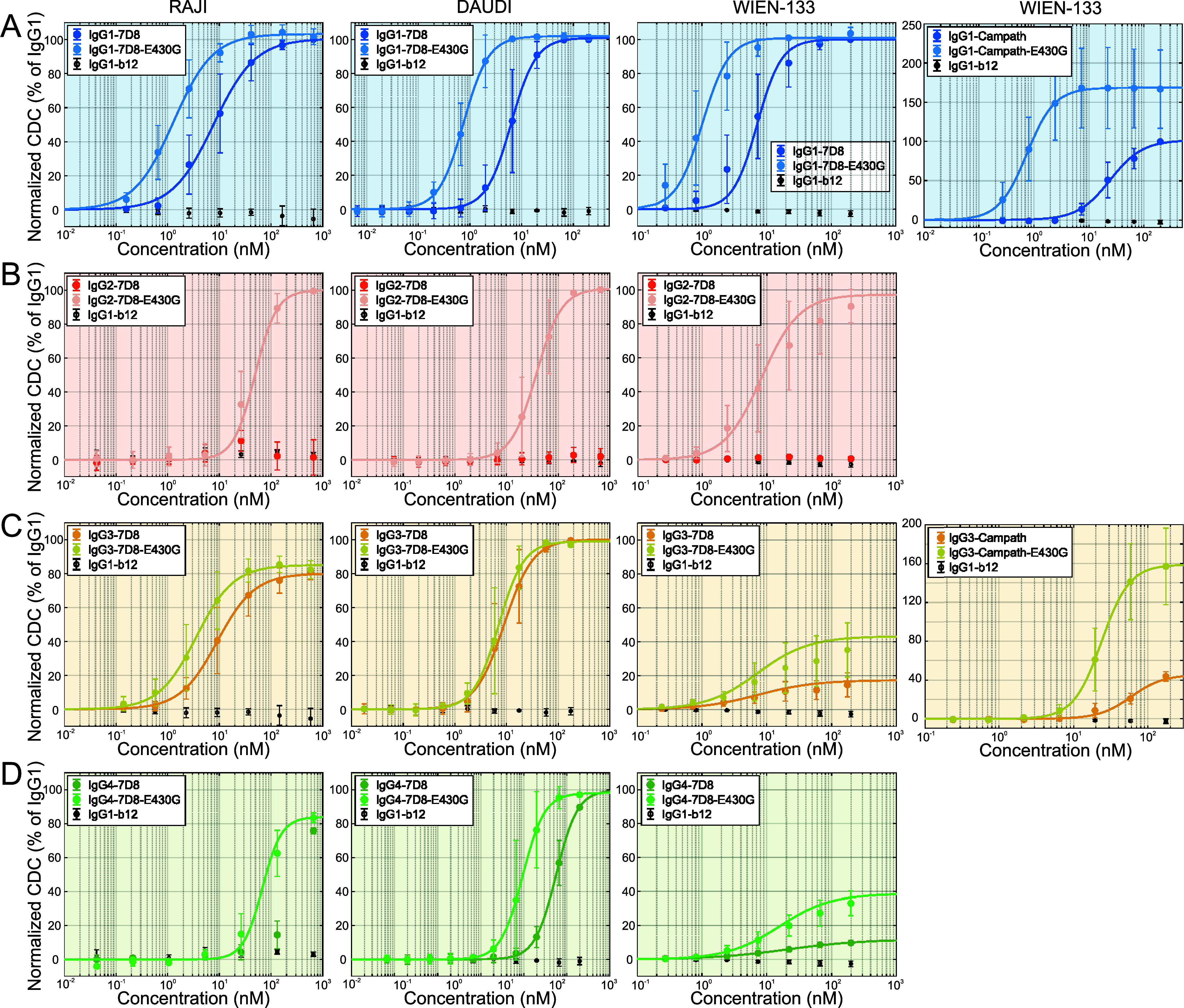
Complement activation by different IgG subclass variants on different lymphoma cell lines and antigenic targets. (*A*) CDC of anti-CD20 antibodies IgG1-7D8 and IgG1-7D8-E430G applied to RAJI, DAUDI, and WIEN-133 cells (panel 1 to 3). Anti-CD52 antibodies IgG1-CAMPATH and IgG1-CAMPATH-E430G applied to WIEN-133 cells (panel 4). (*B*) CDC of anti-CD20 antibodies IgG2-7D8 and IgG2-7D8-E430G applied to RAJI, DAUDI, and WIEN-133 cells. (*C*) CDC of anti-CD20 antibodies IgG3-7D8 and IgG3-7D8-E430G applied to RAJI, DAUDI, and WIEN-133 cells (panel 1 to 3). Anti-CD52 antibodies IgG3-CAMPATH and IgG3-CAMPATH-E430G applied to WIEN-133 cells (panel 4) (*D*) CDC of anti-CD20 antibodies IgG4-7D8 and IgG4-7D8-E430G applied to RAJI, DAUDI, and WIEN-133 cells. Solid lines are fits of dose–response curves to the data; EC50 values are displayed in Table 1.

First, we assessed the efficacy of IgG1-4 and corresponding E430G Fc point mutants, which increased oligomerization and CDC of IgG1 (17, 19, 22), in CDC assays involving different lymphoma cell lines (Fig. 1 and Table 1). CDC of human Burkitt’s lymphoma RAJI and DAUDI cells by human anti-CD20 IgG-7D8 was comparably efficient for IgG1-7D8 (Fig. 1*A*) and IgG3-7D8 (Fig. 1*C*), whereas no CDC was induced by IgG2-7D8 (Fig. 1*B*) and was only detected at highest IgG concentrations in case of IgG4-7D8 without reaching the maximum value of IgG1-7D8 (Fig. 1*D*). Application of anti-CD20 IgG-7D8 subclass variants to WIEN-133 cells yielded comparable EC50 values for CDC albeit maximum lysis was markedly reduced for IgG3-7D8 and IgG4-7D8, down to ~20% as compared to their IgG1 variant. Introduction of the E430G mutation into the anti-CD20 IgGs resulted in significantly lowered EC50 values for lysis by IgG1-7D8-E430G in all three cell lines, and strongly enhanced CDC induced by IgG2-7D8-E430G and IgG4-7D8-E430G, both reaching the EC50 values of IgG1-7D8 and IgG3-7D8. IgG3-7D8-E430G, on the other hand, exhibits only modestly improved EC50 values for CDC of RAJI and DAUDI cells compared to IgG3-7D8, and an improved maximum lysis (40% of the IgG1-7D8) on WIEN-133 cells at a similar EC50 value as IgG3-7D8. Applying human anti-CD52 IgG1-CAMPATH and IgG3-CAMPATH to WIEN-133 (human Burkitt’s lymphoma) cells induced CDC at higher EC50 values as compared to the respective anti-CD20 antibodies, and IgG3-CAMPATH reached only 30% of the maximum lysis of IgG1-CAMPATH. Introduction of the E430G mutation into anti-CD52 IgGs resulted in a potent increase of CDC by IgG1-CAMPATH-E430G (~35-fold lowered EC50 and 60% increased maximum lysis) and a significant improvement of CDC by IgG3-CAMPATH-E430G (~3-fold lowered EC50 and maximum lysis level similar to IgG1-CAMPATH). CDC thus depended on the subclass, with IgG1 being the most effective, closely followed by IgG3 in all cell lines tested, while IgG2 and IgG4 hardly induced CDC. The E430G point mutation significantly lowered EC50 values for CDC throughout all tested cell lines and IgG subclasses, suggesting that comparable to its effect on IgG1 oligomerization, it increases oligomerization of IgG2-4.

**Table 1.** EC50 (antibody concentration inducing half-maximal lysis) values for CDC of antibody-opsonized cells obtained from fitting a dose–response curve of the Hill equation type, $\frac{A}{1+{\left(EC50/C_{\mathrm{IgG}}\right)}^{m}}$, with A being the maximal response, EC50 being the half maximal effective concentration, and m being the Hill coefficient, to the CDC data, Fig. 1

EC50 (nM)	RAJI	DAUDI	WIEN
IgG1-7D8	**5.6** (4.1, 7.0) n = 9	**6.3** (4.7, 7.9) n = 3	**7.2** (6.3, 8.1) n = 3
IgG1-7D8-E430G	**1.0** (0.8, 1.2) n = 9	**0.8** (0.6, 0.9) n = 3	**1.0** (0.9, 1.2) n = 3
IgG1-CAMPATH			**24.2** (16.9, 31.5) n = 5
IgG1-CAMPATH-E430G			**0.7** (0.4, 1.1) n = 5
IgG2-7D8	**n.s.**	**n.s.**	**n.s.**
IgG2-7D8-E430G	**4.9** (3.7, 6.1) n = 3	**3.8** (2.8, 4.7) n = 3	**8.3** (6.1, 10.5) n = 3
IgG3-7D8	**5.6** (4.0, 7.2) n = 9	**9.0** (6.1, 11.8) n = 3	**7.3** (1.1, 13.5) n = 3
IgG3-7D8-E430G	**3.2** (2.5, 3.9) n = 9	**7.0** (5.1, 8.9) n = 3	**7.3** (3.0, 11.7) n = 3
IgG3-CAMPATH			**59.6** (48.2, 71.0) n = 3
IgG3-CAMPATH-E430G			**23.7** (15.3, 32.1) n = 3
IgG4-7D8	**n.s.**	**56.4** (44.4, 68.5) n = 3	**20.9** (2.8, 38.9) n = 3
IgG4-7D8-E430G	**6.8** (5.4, 8.2) n = 3	**9.4** (6.1, 12.7) n = 3	**15.5** (8.2, 22.7) n = 3

Values in brackets are 95% CI.

### Antigen-dependent Oligomerization Naturally Occurs in all IgG Subclasses.

**Fig. 2.**
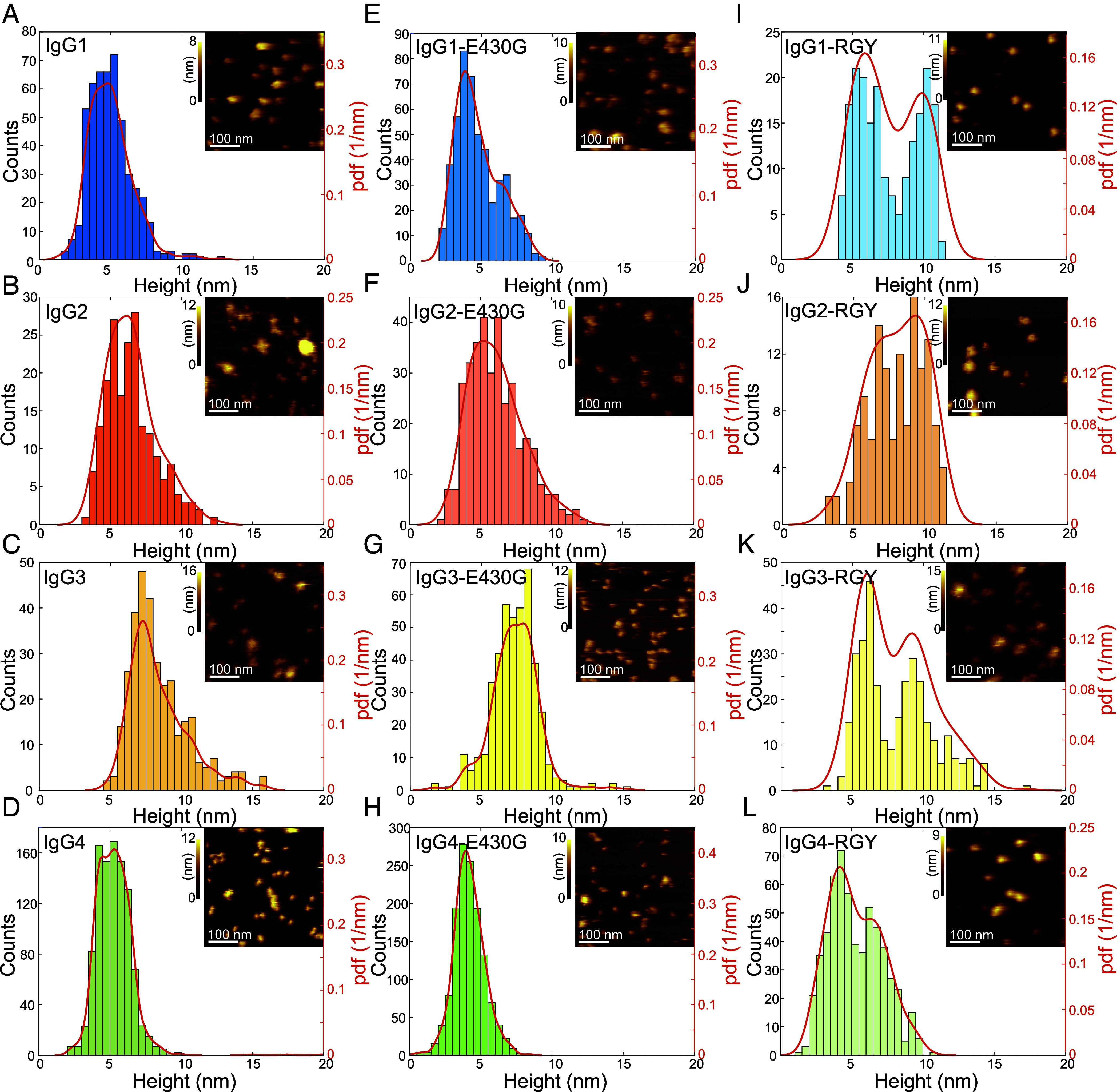
HS-AFM height assessment of different IgG variants bound to antigenic surfaces. (*A*–*D*) Height distributions (histograms and probability density functions) of IgG1-DNP, IgG2-DNP, IgG3-DNP and IgG4-DNP bound to DNP-SLBs. (*E*–*H*) Height distributions of IgG1-DNP-E430G, IgG2-DNP-E430G, IgG3-DNP-E430G, and IgG4-DNP-E430G bound to DNP-SLBs. (*I*–*K*) Height distributions of IgG1-DNP-RGY, IgG2-DNP- RGY, IgG3-DNP-RGY, and IgG4-DNP- RGY bound to DNP-SLBs. Insets represent typical HS-AFM images of the respective IgG variant bound to DNP-SLBs.

Given that the E430G point mutation that increased oligomerization and CDC of IgG1 (17, 19, 22) similarly leads to increased CDC of IgG2-4 E430G variants, we hypothesize that IgG oligomerization is inherent to all subclasses and governs their ability to stably bind and activate C1. We thus prepared supported lipid bilayers (SLB) containing 5% dinitrophenyl (DNP)-labeled lipids (17, 19) to characterize the ability of different IgG subclass variants and mutants to form IgG oligomer populations on antigenic membranes in HS-AFM experiments. While incubation with 33 nM DNP-unspecific IgGs for 5 min did not result in any binding, incubation with IgG1-DNP, IgG2-DNP, IgG3-DNP, and IgG4-DNP under similar conditions generated sparse distributions of differently sized assemblies on top of the SLBs, characteristic for IgG oligomer distributions as previously found for IgG1 (17, 19). Unlike the other subclasses, addition of IgG2 frequently led to detachment of the SLBs from the mica support so that only smaller IgG2-bearing lipid patches that remained firmly attached to the support could be analyzed. We mainly observed isolated, likely bivalently bound, IgGs with heights of 4 to 7 nm (IgG1, IgG2, and IgG4) and 5-12 nm (IgG3) and smaller fraction of higher-order IgG assemblies with heights ranging up to 11 nm (IgG1, IgG2, and IgG4) and 15 nm (IgG3), respectively (Fig. 2 *A*–*D*). The E430G single point mutation only had a recognizable effect on the height distribution of IgG1-DNP-E430G with the second height population between 6 and 10 nm becoming more prominent as compared to IgG1-DNP (Fig. 2*E*). For the other subclasses, no such change in height distribution was apparent (Fig. 2 *F*–*H*). The triple mutants IgG1-DNP-RGY, IgG2-DNP-RGY, IgG3-DNP-RGY, and IgG4-DNP-RGY (E345R, E430G, and S440Y), which were previously shown to efficiently associate into IgG hexamers in solution (1, 21, 23), exhibited the largest fraction of large IgG assemblies as evident from the broadening of the height distributions and the appearance of a second, higher peak for all four subclasses (Fig. 2 *I*–*L*). Interestingly, the IgG3 variants always contained a fraction that protruded further (up to 15 nm) from the antigenic membranes and then the respective IgG1, IgG2, and IgG4 variants (up to 10 to 11 nm). They also exhibited a higher resistance to increased HS-AFM imaging forces than the other three IgG subclasses, which could be more easily removed from the membranes when intentionally lowering the HS-AFM setpoint amplitude, potentially indicating bivalent antigen attachment of IgG3 molecules within oligomers.

### IgG3 Oligomers are Structurally Distinct from Other IgG Subclasses.

**Fig. 3.**
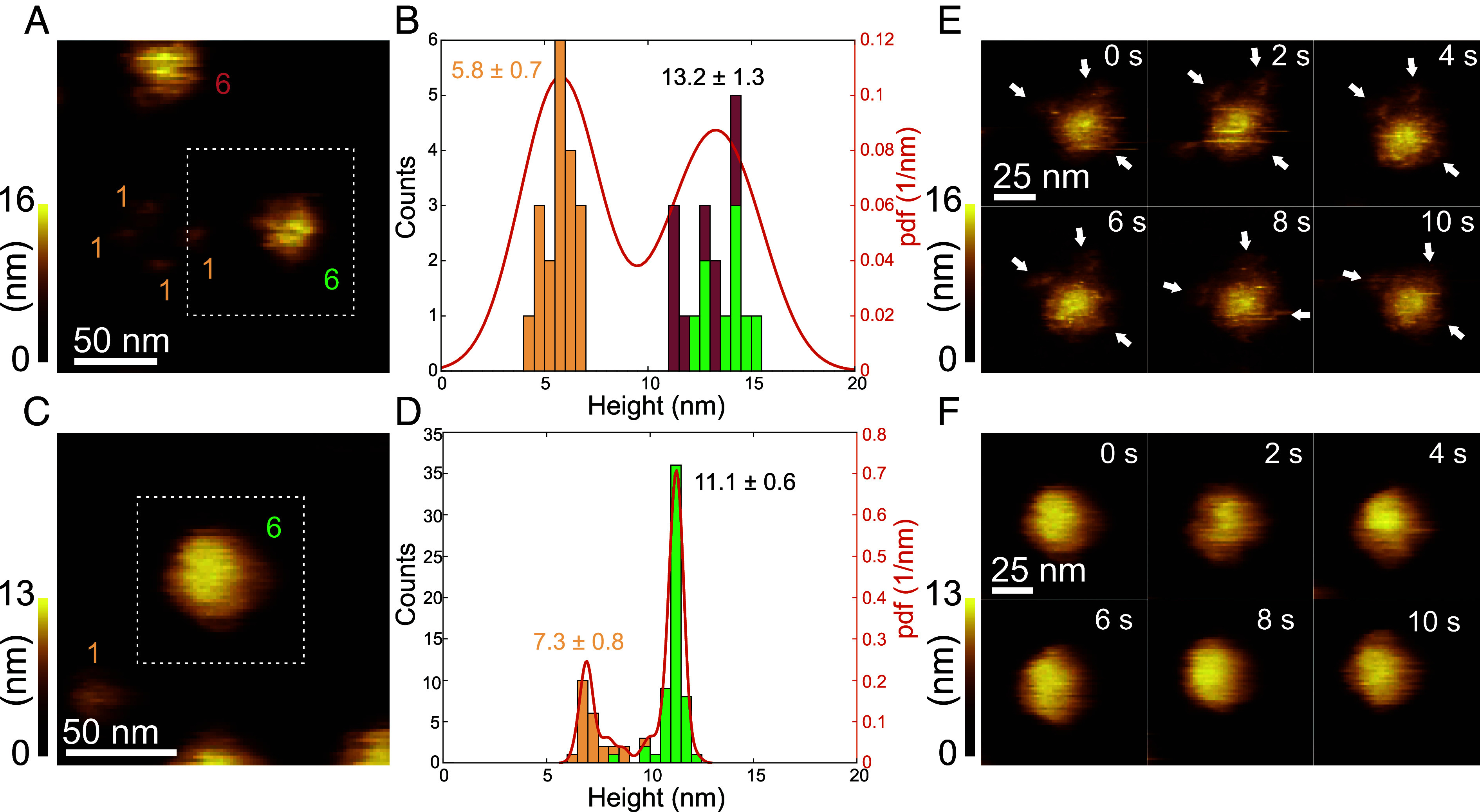
Structural comparison of IgG3 and IgG1 hexamers based on higher-resolution HS-AFM images. (*A*) First frame of HS-AFM Movie S1 of two IgG3 hexamers and four IgG3 monomers bound to a DNP-SLB. (*B*) Height histogram generated from height over time recordings of the oligomers in Movie S1. Contributions of the respective oligomers are color coded according to (*A*). Numbers correspond to means ± SD over all image frames and individual particles, respectively. (*C*) First frame of HS-AFM Movie S3 of an IgG1 hexamer and one IgG1 monomer bound to a DNP-SLB. (*D*) Height histogram generated from the height over time of the oligomers in Movie S3. Contributions of the respective oligomers are color coded according to (*C*). (*E*) High-resolution images of an individual IgG3 hexamer [dashed area from (*A*)] taken from HS-AFM Movie S4. Additional smaller structures surrounding the central Fc platform are indicated by arrows. (*F*) High-resolution images of an individual IgG1 hexamer [dashed area from (*C*)].

To further examine these topographical differences, we obtained higher-resolution images of IgG3-DNP assemblies on antigenic membranes as exemplified in Fig. 3*A*, taken from HS-AFM Movie S1. Judging from the lateral size, symmetry, and mobility of the respective assembly, we identified them as monomers and hexamers as indicated in Fig. 3*A*. Tracking these IgG oligomers over time enabled us to evaluate their heights in each image frame, from which we again generated height histograms (Fig. 3*B*). The IgG3-DNP monomers recorded in this HS-AFM movie were characterized by a height of 5.8 nm that fluctuated by ±0.7 nm during the experiment, whereas the hexamers protruded on average 13.2 nm from the antigenic membrane and fluctuated by ±1.3 nm (mean ± SD overall image frames and individual particles, respectively). *SI Appendix*, Fig. S1*A* depicts the first HS-AFM image frame of Movie S2, another example of IgG3 oligomers bound to an antigenic membrane; however, additionally to monomers and a hexamer, a tetramer was followed and analyzed over time. While height histograms of monomers and hexamers were consistent with the respective distributions from Fig. 3*B*, the height distribution of the tetrameric IgG3 was located in between the monomers and the hexamer exhibiting an average height of 9.4 nm and fluctuations of ±1.7 nm. Repeating these experiments and height analysis for IgG1 (Fig. 3*C* and Movie S3) resulted in much narrower distributions with average heights of 7.3 nm and 11.1 nm, and height fluctuations of ±0.8 nm and ±0.6 nm for IgG1 monomers and hexamers, respectively. These experiments suggest that the height distributions compiled from a large ensemble of antigenic membrane-bound IgG assemblies as depicted in Fig. 2 include overlapping contributions from differently sized IgG oligomers with the lower heights stemming mainly from IgG monomers and the larger ones from the IgG hexamers. Zooming further in on IgG3 hexamers (Fig. 3*E* taken from Movie S4 and *SI Appendix*, Fig. S1*C*) clearly revealed a central platform consisting of six Fc domains (18) with an overall shape and diameter as previously found for IgG1 hexamers (17), but with smaller portions that laterally protruded below the platform (indicated by arrows) that were not found in case of IgG1 hexamers (Fig. 3*F*). These smaller parts slowly changed their position around the central platform over time; however, the fact that they could be resolved by the HS-AFM tip suggests that they represent antigen-bound IgG3 Fabs since unbound Fabs as present in IgG1 hexamers would be subject to much faster diffusional motion far beyond the time-resolution of the HS-AFM (24).

### Abundance of Higher IgG Oligomers Depends on Subclass and Is Increased by E430G Point Mutation.

**Fig. 4.**
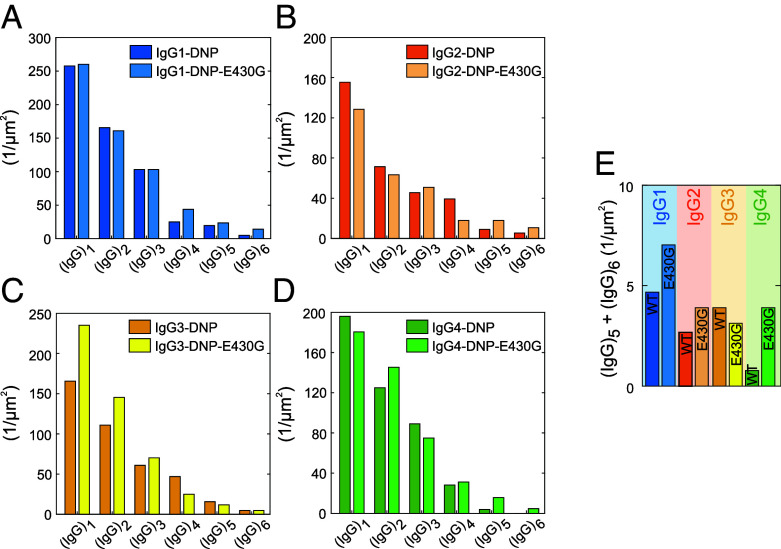
Oligomer distributions of anti-DNP IgG1-4 and the respective E430G point mutants bound to DNP-SLBs examined by HS-AFM. (*A*) IgG1-DNP and IgG1-DNP-E430G. (*B*) IgG2-DNP and IgG2-DNP-E430G. (*C*) IgG3-DNP and IgG3-DNP-E430G. (*D*) IgG4-DNP and IgG4-DNP-E430G. (*E*) Sum of IgG pentamer and hexamer densities for each IgG variant.

HS-AFM imaging further allowed us to compile quantitative oligomer distributions for each subclass variant on DNP-SLBs. The molecules were therefore scanned in a nondisrupting manner to gauge their number, height, and shape, followed by an increase in scanning force applied by the HS-AFM cantilever tip to dissociate the oligomers into their constituent individual IgGs (17, 19). Geometric parameters and oligomer decay patterns were combined to assign each IgG assembly its oligomeric state (Fig. 4). Characteristic oligomer distributions were found for all investigated IgGs, with smaller oligomers occurring more frequently than large ones. While IgG1-DNP, IgG2-DNP, and IgG3-DNP already formed oligomers up to hexamers under the chosen conditions (Fig. 4 *A*–*C*), only a few pentamers were detected in the case of IgG4-DNP (Fig. 4*D*). Introduction of the E430G point mutation increased the oligomerization propensity of IgG1, IgG2, and IgG4 leading to a significantly higher amount of pentamers and hexamers relative to the respective parental IgG, while the abundance of IgG3-DNP-E430G pentamers and hexamers stayed mostly at the same level as IgG3-DNP (Fig. 4*E*).

### C1 Recruitment Efficiency Correlates with IgG Oligomerization Propensity.

**Fig. 5.**
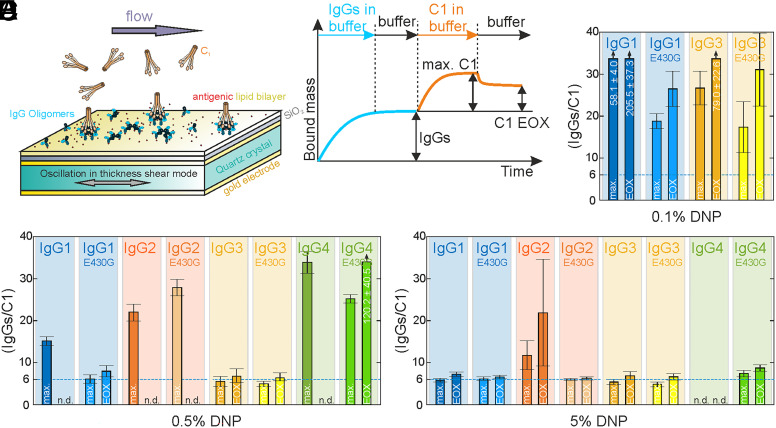
Impact of antigen surface density on C1 recruitment efficiencies of different IgG subclasses and E430G point mutants. (*A*) Illustration of a QCM experiment with C1 molecules in the running buffer over an IgG opsonized DNP-SLB prepared on a SiO2-coated gold electrode of an oscillating quartz crystal. (*B*) Schematic QCM sensorgram of a typical experiment. For calculation of the recruitment efficiencies, the IgG densities were divided by either the max. C1 binding level or the level at the end of the experiment (EOX). (*C*) C1 recruitment efficiencies obtained for a medium antigen density of 0.5 mol% DNP-labeled lipids in the DNP-SLB. (*D*) C1 recruitment efficiencies of IgG1 and IgG3 variants obtained for a low antigen density of 0.1 mol% DNP-labeled lipids in the DNP-SLB. (*E*) C1 recruitment efficiencies of IgG1 and IgG3 variants obtained for a high antigen density of 5 mol% DNP-labeled lipids in the DNP-SLB. Depicted recruitment efficiencies are means ± SD.

To address the question of whether the observed oligomerization propensity is directly related to complement C1 fixation, we characterized the ability of the different IgG subclasses and E430G point mutants to recruit C1 to antibody-opsonized antigenic membranes in quartz crystal microbalance (QCM) experiments (19) employing the same DNP-SLBs used in HS-AFM but generated on a QCM sensor chip (Fig. 5*A*). Monitoring the change of oscillation frequency of the DNP-SLB covered SiO_2_-coated quartz crystal, which is proportional to the change in bound mass due to association of molecules out of the constant buffer flow above the chip surface yields characteristic binding curves for lipids, IgGs and subsequently added complement proteins (*SI Appendix*, Fig. S2). We prepared three DNP-SLBs differing in antigen densities (0.1, 0.5, and 5% DNP) to which the respective IgG variant was allowed to associate for a defined timespan. After establishing a certain IgG density (~2, 3, and 7 × 10^3^ IgGs/µm^2^, respectively) and removal of solution phase IgGs, C1 was added to the running buffer and the binding signal was followed over time until the end of the dissociation phase in which C1 was again removed from the running buffer (*SI Appendix*, Fig. S3 *A* and *B*). To better compare the respective C1 binding levels between different experiments, we introduced the ratio of bound IgGs to C1 molecules evaluated at the maximum (max.) C1 level and the final C1 level at the end of the experiment (EOX), respectively (Fig. 5*B*). Importantly, these ratios of average IgGs needed to recruit a single C1 molecule to our antigenic membranes define the density of IgGs needed to generate a binding site for a single C1; however, they are not necessarily reflecting the number of IgGs actually interacting with a C1 molecule (limited by max. six gC1q binding heads per C1). Still, the recruitment efficiency never went below ~6 IgGs/C1 which hints at the optimal (most stable) complex size being a C1 molecule bound to an IgG hexamer. The comparison of these ratios resulting from max. C1 and EOX C1 enable evaluation of the stability of the established IgG–C1 complexes and their variation with subclass and mutant variants.

At a medium antigen density (0.5% DNP, Fig. 5*C*), C1 recruitment was most efficient for IgG3-DNP, followed by IgG1-DNP, IgG2-DNP, and IgG4-DNP. Stable binding with close to no dissociation was only observed for IgG3-DNP (reflected by a comparable IgGs/C1 ratio at max. and EOX). C1 dissociated completely from IgG1-DNP, IgG2-DNP, and IgG4-DNP (n.d.). Importantly, IgG3-DNP exhibited the lowest IgGs/C1 ratios suggesting that on average ~six IgG3s were needed to stably bind a single C1 molecule to the antigenic membrane. The E430G point mutation that increased oligomerization of IgG1-DNP-E430G, IgG2-DNP-E430G, and IgG4-DNP-E430G but left IgG3-DNP -E430G oligomerization unaltered (Fig. 4) had a similar effect on C1 binding: It significantly lowered the average number of IgG1-DNP-E430G molecules needed to stably bind a single C1 to ~8 and increased the max. C1 binding of IgG4-DNP-E430G (thus lowering the respective IgGs/C1 ratio), although to a much lesser extent. IgG3-DNP-E430G did not perform significantly differently from the highly efficient IgG3-DNP, suggesting that similarly to oligomerization, C1 binding was not affected by the mutation. To check whether this still holds true for antigen densities typically observed on tumor cell lines (cf. *Materials and Methods*), we repeated these experiments for IgG1 and IgG3 (and respective E430G variants) using 0.1% DNP SLBs (Fig. 5*D*). Compared to 0.5% DNP, C1 recruitment and stable binding were vastly reduced for IgG1-DNP, less so for IgG1-DNP-E430G; however, unlike at 0.5% DNP, IgG3-DNP-E430G was significantly more efficient (~2.5 times) than IgG3-DNP (max. and EOX) in recruiting C1.

Repeating these experiments at higher antigen densities (Fig. 5*E*) improved C1 recruitment and stable binding for IgG1-DNP, which could no longer be distinguished from IgG1-DNP-E430G, established a stable bound C1 population on IgG2-DNP, and enabled highly efficient C1 recruitment to IgG2-DNP-E430G. Surprisingly, unlike at lower antigen densities (and accordingly lower IgG4-DNP densities of ~3 × 10^3^ IgGs/µm^2^) where at least transient C1 binding was detectable on IgG4-DNP, high IgG4-DNP densities (~7 × 10^3^ IgGs/µm^2^) resulting from high antigen densities were found to render antigen-bound IgG4-DNP molecules incapable of interacting with C1, presumably because molecular crowding caused by a high density of bivalently bound IgG4-DNP monomers effectively impedes the formation of higher oligomers capable of stable C1 binding. In contrast, IgG4-DNP-E430G was found to exhibit highly efficient C1 recruitment, with levels comparable to the E430G variants of the other three subclasses. Increasing the antigen density did not further improve C1 recruitment efficiency and stability for any IgG variant below ~six IgGs/C1 suggesting that the vast majority of antigen-bound IgGs are arranged in hexamers at this highest antigen density. Notably, for each IgG variant, a certain minimal antigen density is needed to reach this stable level of six IgGs/C1, whereas the E430G point mutation seems to compensate for low antigen densities across subclasses. We have previously (20) noticed that C1 binds stronger to all subclasses than the C1q recognition molecule alone. We hypothesized that C1r_2_s_2_ proteases enhance the stability of the C1q–IgG complexes by fixing the collagen arms, thereby aligning the gC1q domains in a hexagonal platform that favors binding to IgG oligomers. To further substantiate this hypothesis, we have repeated the above QCM experiments with C1q instead of C1 and indeed found that C1q binding is less efficient in all settings except for IgG1-DNP-E430G which was similarly effective in recruiting C1 and C1q on medium and high antigen densities (*SI Appendix*, Fig. S3 *C*–*F*).

### Complex Kinetics of C1/C1q Binding to IgG Oligomers.

Differences in complement activation between IgG subclasses are often associated with their respective monovalent affinities for the gC1q headpieces (4) and their relative hinge flexibilities, which may modulate the accessibility of gC1q binding sites on the IgG Fc domain through shielding effects caused by the Fabs (25). However, as shown above, complement binding and CDC clearly correlates with IgG oligomerization on an antigenic surface and can be maximized by introduction of a mutation that solely enhances IgG oligomerization, not the affinity for gC1q. It is thus likely that the extent of multivalency of the interaction between C1 and IgG oligomers is the main prerequisite for complement activation, while the monovalent affinity of the respective IgG subclass for gC1q plays a rather subordinate role.

**Fig. 6.**
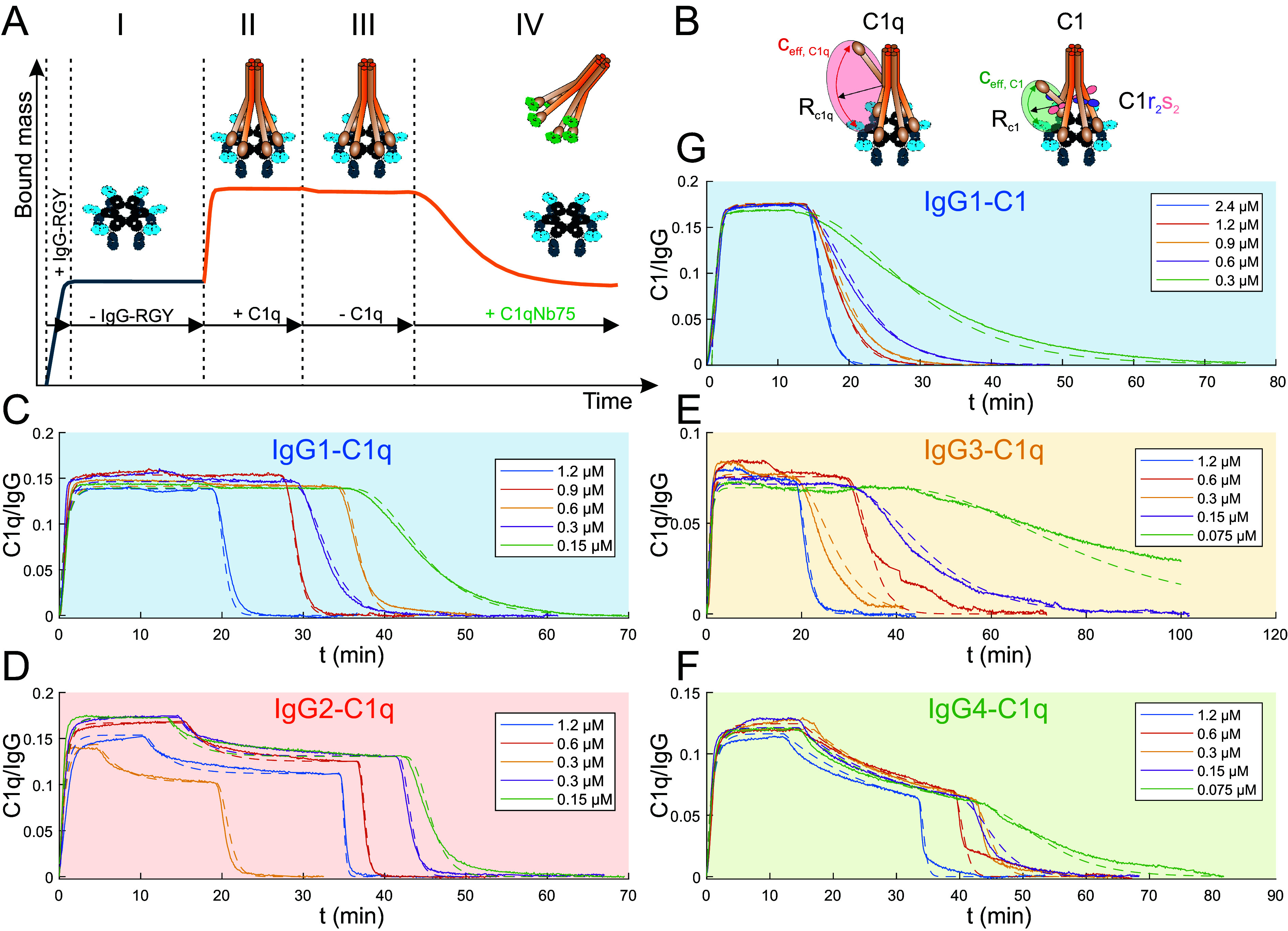
Kinetic analysis of complement C1q and C1 binding to IgG oligomers of different subclasses. (*A*) QCM sensorgram of a typical competition experiment, exemplified by IgG1-DNP-RGY and C1q. After establishing a certain IgG-RGY density on the DNP-SLBs (I) C1q or C1 was added to the QCM running buffer resulting in saturated binding (II; cf. *SI Appendix*, Fig. S4). Removal of C1q/C1 from the running buffer typically induced only minor dissociation (III). Addition of the high-affinity gC1q binding C1qNb75 nanobody induced complete dissociation of C1q/C1 from the underlying IgG oligomers. (*B*) Sketch of C1q/C1–IgG hexamer complex with an unbound gC1q head. The presence of C1r_2_s_2_ reduces the effective collagen-arm length thereby increasing the local gC1q concentration in C1 as compared to C1q. (*C*) C1qNb75 nanobody concentration series applied to IgG1-DNP-RGY–bound C1q (solid lines). Significant contributions of IgG1-RGY oligomers to C1q binding: 86.2% in hexamers. (*D*) C1qNb75 nanobody concentration series applied to IgG2-DNP-RGY–bound C1q (solid lines). Significant contributions of IgG2-RGY oligomers to C1q binding: 72.2% in hexamers and 18.3% in trimers. (*E*) C1qNb75 nanobody concentration series applied to IgG3-DNP-RGY–bound C1q (solid lines). Significant contributions of IgG3-RGY oligomers to C1q binding: 44.1% in hexamers. (*F*) C1qNb75 nanobody concentration series applied to IgG4-DNP-RGY–bound C1q (solid lines). Significant contributions of IgG4-RGY oligomers to C1q binding: 30.6% in hexamers and 22.6% in trimers. (*G*) C1qNb75 nanobody concentration series applied to IgG1-DNP-RGY–bound C1 (solid lines). Significant contributions of IgG1-RGY oligomers to C1 binding: 73.3% in hexamers and 5.7% in dimers. Dashed lines in (*C*)–(*G*) represent fits to our mechanistic model (*SI Appendix*, Fig. S5).

To determine affinities and kinetic rate constants of the interactions between gC1q headpieces within C1/C1q and IgG monomers within IgG hexamers, we employed the triple mutants IgG1-DNP-RGY, IgG2-DNP-RGY, IgG3-DNP-RGY, and IgG4-DNP-RGY which were shown to efficiently associate into IgG hexamers in solution (1, 21, 23) to generate an IgG oligomer distribution with a high relative surface density of IgG hexamers. After incubation of the respective IgG-DNP-RGY variant on DNP-SLBs generated on a QCM chip and removal of solution phase IgGs, C1q was added to the running buffer and the binding signal was followed over time [Fig. 6*A*, exemplified for IgG1-DNP-RGY (I)]. Binding quickly reached saturation (II) (*SI Appendix*, Fig. S4) but only minor dissociation, likely from smaller oligomers that form upon antigen binding, was observed when C1q was removed from the running buffer (III). No dissociation rates could be obtained from such curves because even though gC1q within a multivalently bound C1q presumably dissociated from IgG Fcs occasionally, immediate rebinding stabilized the hexavalent IgG-RGY–C1q complexes. To nevertheless induce dissociation, we performed competition experiments in which a high-affinity competitor [nanobody C1qNb75 (26)] for gC1q was introduced into the QCM flow cell, which strongly binds to transiently unbound gC1q heads, thereby preventing rebinding and eventually leading to complete dissociation of C1q molecules from IgG-RGY hexamers (Fig. 6 *A, IV*). To analyze these data, we developed a corresponding kinetic model of C1/C1q interacting with IgG oligomers (monomers to hexamers) in the absence (Fig. 6 *A, II* and *III*) and presence (Fig. 6 *A, IV*) of a competitor binding to gC1q heads and thus blocking their interactions with IgG Fcs (*SI Appendix*, Fig. S5). Since the association and dissociation rate constants (k_on,comp_ and k_off,comp_, respectively) of the competitor are known (26), only three additional parameters are required to describe these interactions: The association and dissociation rate constants of the monovalent IgG-Fc–gC1q interactions (k_on,gC1q_ and k_off,gC1q_, respectively) and a parameter that describes the effective concentration (c_eff_) of an unbound gC1q head within an oligomer-bound C1q molecule (Fig. 6*B*). We performed competitor concentration series for each IgG subclass and fitted the model to the QCM data to extract the three unknown parameters, where k_on,gC1q_ and k_off,gC1q_ were fit locally for each subclass, and c_eff_ globally over all subclasses (Fig. 6 *C*–*F* and Table 2). The resulting equilibrium dissociation constants (K_D_) were collectively found to be ~10 times lower than previously reported, suggesting that individual IgGs within an oligomer configuration bind C1q with ~10 times higher affinity as compared to monovalent IgGs in solution (5). The IgG3–gC1q interaction had the lowest K_D_ (4.1 µM), followed by IgG1 (8.0 µM), but different from what was previously found, followed first by IgG4 (11.3 µM) and only then IgG2 (20.0 µM). Repeating this experimental strategy for the interaction between IgG1 and C1 instead of C1q allowed us to quantify the difference between C1 and C1q binding to IgG oligomers. While the association and dissociation rate constants of gC1q heads within C1 and C1q molecules should not be affected by the addition of C1r_2_s_2_ proteases, we have hypothesized that the C1r_2_s_2_ proteases in C1 enhance the stability of C1q–IgG complexes by confining the motional freedom of gC1q heads to a geometry that favors binding to IgG oligomers, which is equivalent to an increase of the effective concentration (c_eff, C1_) in our kinetic model (Fig. 6*B*). We have thus used the association and dissociation rate constants obtained from the IgG1-RGY–C1q interaction (Fig. 6*A*) and only adjusted the effective concentration (c_eff, C1_) to fit the IgG1-RGY–C1 competition concentration series (Fig. 6*G* and Table 2). We found that the presence of C1r_2_s_2_ proteases increases the effective concentration of an unbound gC1q head within an oligomer-bound C1q molecule from 0.4 to 1.5 mM, which corresponds to a reduction of the effective arm length to which the gC1q head is linked within the C1q molecule from 17.7 to 11.7 nm, assuming a spherical volume.

**Table 2.** Parameters obtained from fitting the model (*SI Appendix*, Fig. S5) to the data in Fig. 6 *B*–*F*

	IgG1	IgG2	IgG3	IgG4
k_on, gC1q_ (M^-1^s^-1^)	**4.2** (3.2, 5.5) × **10^4^**	**5.1** (3.2, 8.3) × **10^4^**	**4.5** (2.4,10.2) × **10^4^**	**5.0** (3.3, 9.3) × **10^4^**
k_off, gC1q_ (s^-1^)	**0.33** (0.22, 0.65)	**1.01** (0.26, 1.74)	**0.18** (0.09, 0.48)	**0.56** (0.45, 1.0)
K_D_ (µM)	**7.9** (5.2, 12.1)	**19.8** (4.8, 40.4)	**4.0** (2.6, 6.2)	**11.2** (6.7, 18.1)
C_eff, C1q_ (mM)	**0.43** (0.26, 0.81)			
C_eff, C1_ (mM)	**1.9** (1.7, 2.0)			
R_C1q_ (nm)	**17.7** (14.3, 20.9)			
R_C1_ (nm)	**10.8** (10.6, 11.2)			

Effective arm lengths were calculated from the effective concentrations by modeling the respective volume as a sphere. Values in brackets are 95% CI.

### Functional Affinities of C1(q) to IgG1-4 Oligomers.

Extending the expression for the functional affinity (avidity) of two monovalent interactions that are linked via a flexible linker molecule (19, 27), we can estimate the functional affinity between C1(q) and IgG subclass oligomers of different sizes n = 1 to 6 via

[1] $$K_{D,n}=\frac{K_{D}}{6}{\left(\frac{K_{D}}{c_{\mathrm{eff}}}\right)}^{n-1}.$$

**Fig. 7.**
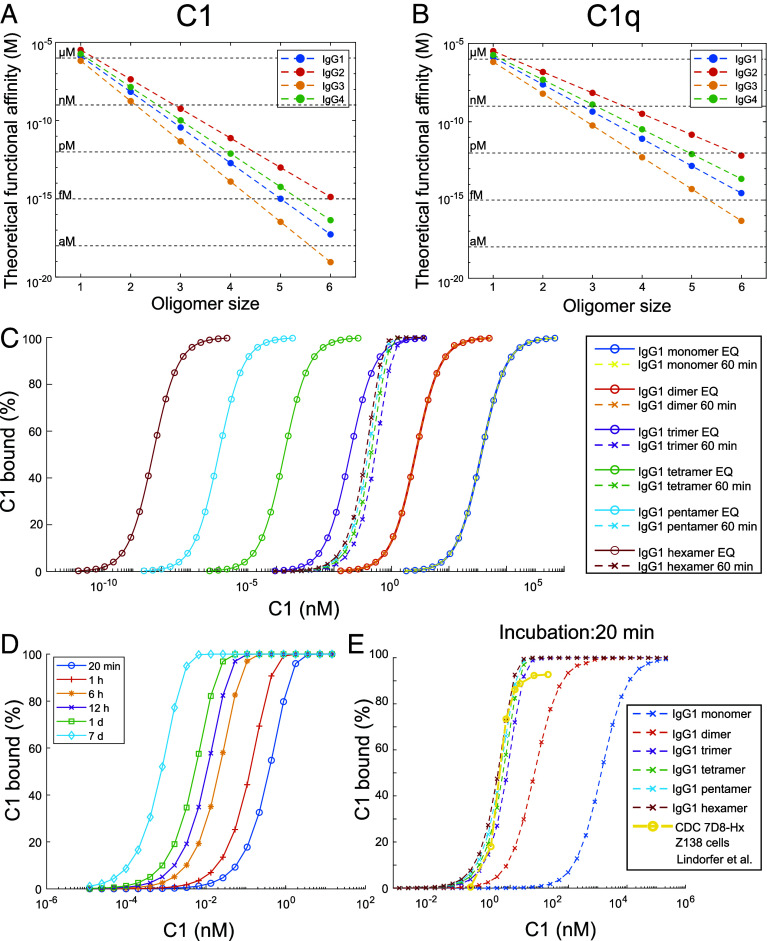
C1/C1q binding to differently sized IgG subclass oligomers. (*A* and *B*) Comparison of functional affinities of C1 (*A*) and C1q (*B*) to differently sized IgG subclass oligomers determined from kinetic simulations (until equilibrium is reached, dots) and from the thermodynamic approach (dashed lines), using the parameters from Table 2, respectively. (*C*) Concentration dependency of C1 binding to IgG1 monomers to hexamers at equilibrium (circles) and after an incubation time of 60 min (crosses). (*D*) Influence of incubation time on C1 binding to IgG1 hexamers. (*E*) Concentration dependency of C1 binding to IgG1 monomers to hexamers after an incubation time of 20 min. Comparison to CDC data of anti-CD20 antibody IgG1-7D8-E430G bound to Z138 cells taken from (28).

Using the subclass-specific K_D_ values and c_eff_ (*SI Appendix*, Table S2 and Fig. 6*B*) thus allows us to derive functional affinity constants for all 4 IgG subclasses and all oligomer sizes (Fig. 7 *A*, *B* for C1 and C1q, respectively). The thermodynamic derivation (Eq. **1**) reproduces the K_D_ values obtained from directly fitting a Langmuir isotherm to the simulation of the concentration-dependent equilibrium binding of C1/C1q to IgG oligomers (Fig. 7*C*, exemplified for C1 binding to IgG1 monomers–hexamers). Importantly, typical incubation times employed experimentally [60 min in (1), 20 min in (28)] in combination with low C1 concentrations in the pM regime do not allow the system to reach equilibrium and thus do not lead to saturating binding of C1 to IgG1 tetramers and higher oligomers (Fig. 7 *C*–*E*).

### IgG Oligomerization and Resulting C1 Binding Govern Complement-dependent Iysis of DNP-coated Vesicles.

**Fig. 8.**
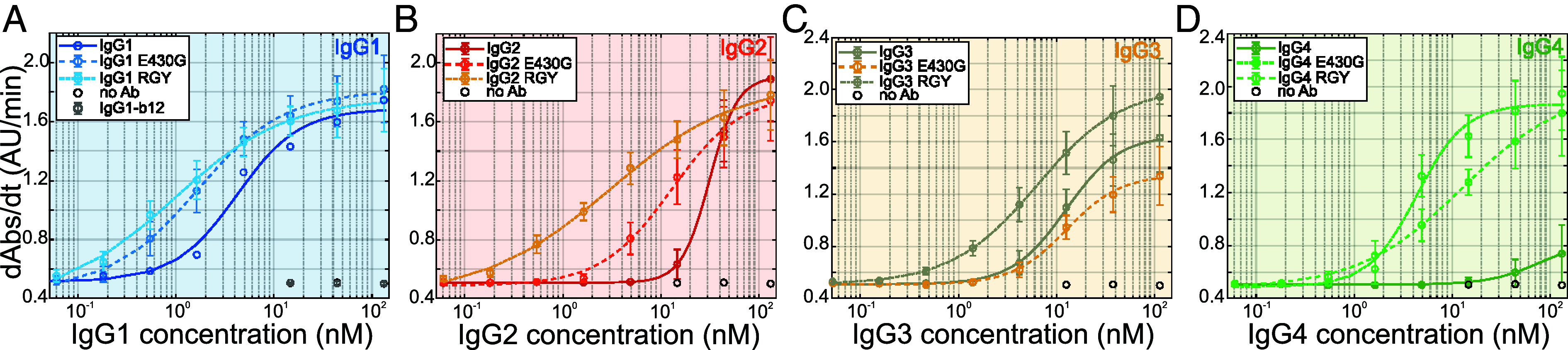
Complement-mediated lysis of DNP-coated liposomes induced by anti-DNP IgG variants. (*A*–*D*) The ability of graded amounts of IgG1-4 variants to induce activation of complement in human serum is assessed by the magnitude of complement-mediated liposome lysis expressed as the change in absorbance at 340 nm (Abs) per second (dAbs/dt). Each data point represents means ± SD from n = 3 independent experiments. Lines are fits of dose–response curves to the data; EC50 values are displayed in Table 3.

To link our observations of subclass-specific IgG oligomerization, complement C1 recruitment, and affinities on DNP-SLBs directly to terminal complement activation, we performed DNP-labeled liposomal vesicle-based complement lysis assays (17) employing the same anti-DNP IgG variants used in our HS-AFM and QCM studies (Fig. 8). The employed vesicles contained 0.5% DNP (29) which corresponds to 2 to 10-fold higher antigen density as compared to CD20 densities on lymphoma cells (cf. *SI Appendix*, *Materials and Methods*). Fitting an agonist/response curve to the vesicle lysis data yielded EC50 values (antibody concentration inducing half-maximal lysis) for every IgG variant (Table 3). EC50 was smallest (4.1 nM) for IgG1-DNP, followed by IgG3-DNP (12.5 nM), IgG2-DNP (31.5 nM), and IgG4-DNP with no significant lysis even at the highest IgG4-DNP concentration. The E430G point mutation lowered the EC50 values of IgG1-E430G-DNP (1.6 nM), IgG2-E430G-DNP (12.8 nM), and IgG4-E430G-DNP (4.4 nM) relative to the respective parental IgG, but the EC50 of IgG3-E430G-DNP (12.6 nM) remained essentially unchanged compared to IgG3-DNP (12.5 nM), which is consistent with our QCM experiments performed at the same medium antigen density of 0.5% DNP (Fig. 5*C*). Triple mutants IgG-RGY-DNP induced strongest complement-dependent lysis in all subclassed except for IgG4-RGY which was found to be less efficient than IgG4-E430G-DNP.

**Table 3.** EC50 (antibody concentration inducing half-maximal lysis) values for lysis of antibody-opsonized DNP-coated vesicles obtained from fitting a dose–response curve of the Hill equation type $Y_{bottom}+\frac{\left(Y_{top}-Y_{bottom}\right)}{1+{\left(EC50/C_{IgG}\right)}^{m}}$, with (*Y_top_* − *Y_bottom_*) being the maximal response, *Y_bottom_* being the background signal, EC50 being the half maximal effective concentration, and m being the Hill coefficient, to the CDC data, Fig. 8

	IgG1	IgG1-E430G	IgG1-RGY
EC50 (nM)	**4.1** (0.5, 7.8)	**1.6** (1.1, 2.1)	**0.9** (0.4, 1.4)
	IgG2	IgG2-E430G	IgG2-RGY
EC50 (nM)	**31.5** (31.1, 31.8)	**12.8** (7.4, 18.2)	**3.0** (1.5, 4.5)
	IgG3	IgG3-E430G	IgG3-RGY
EC50 (nM)	**12.5** (10.5, 14.4)	**12.6** (9.8, 15.5)	**6.3** (5.7, 6.8)
	IgG4	IgG4-E430G	IgG4-RGY
EC50 (nM)	**n.s.**	**4.4** (2.4, 6.3)	**12.6** (9.7, 15.6)

Values in brackets are 95% CI.

## Discussion

The classical pathway of complement activation is a key part of the immune system, aiding in both defense against infections and the regulation of immune responses. Additionally, it is exploited in targeted immunotherapy to eliminate infectious agents, regulatory immune cells, or cancer cells. Its activation depends strongly on the IgG subclass, with IgG1 and IgG3 being able to efficiently trigger activation (30), while IgG2 and IgG4 are usually much less efficient or activate complement only under certain conditions such as IgG2 on high antigen density surfaces like bacterial polysaccharides (20, 31, 32). IgG4, being mostly functionally monovalent in plasma, was reported to form only small immune complexes incapable of complement activation, thereby effectively inhibiting complement activation through competing with IgG1 for targets on cell surfaces (33). The ability to activate complement is associated with the binding affinity of C1q to the respective subclass (5, 30, 34), but also downstream events such as C4b deposition might be differentially affected (18, 30). We have previously shown that antigen-bound IgG1 can exist as a distribution of monomers to hexamers. Only tetramers, pentamers, and hexamers could trigger complement-dependent cytotoxicity (CDC) since at least four gC1q headpieces were required to bind to adjacent subunits within these IgG1 oligomers (7). IgM pentamers with one J-chain and IgM hexamers without J-chain adopt very similar structures, where the distances between residues for gC1q binding are identical to the respective distances in IgG1 hexamers (3) representing similar stereochemical danger patterns recognized by C1q. More recently, cryo-ET images of the C1–IgG3 complex revealed similar Fc platforms with all 6 gC1q headpieces bound to IgG3 hexamers (18). These studies suggest that the binding of only two or three adjacent gC1q headpieces to IgG1 or IgG3 oligomers does not result in sufficient compaction of the C1q arms to induce the conformational rearrangements that allow C1r to activate C1s (2). Given these similarities in C1 binding and activation between IgG1, IgG3, and IgM, we hypothesized that it is primarily an IgG’s ability to oligomerize on antigenic surfaces to form sufficiently large multivalent targets for C1, that ultimately determines whether complement activation is initiated or not.

We here demonstrate that indeed all four IgG subclasses are capable of oligomerization, and that the resulting valency of IgG oligomers determines their capacity to multivalently bind C1 which in turn is directly linked to complement activation and CDC. We first evaluated the potency of anti-CD20 and anti-CD52 IgG subclass variants to kill different tumor cells in CDC assays (Fig. 1 and Table 1). IgG1 and IgG3 were comparably effective in all experimental settings, while IgG2 and IgG4 hardly induced CDC, reproducing the relative CDC efficacies known from the literature (30). The E430G point mutation significantly decreased EC50 values for CDC in all cell lines and for all IgG subclasses, suggesting that it increases IgG2-4 oligomerization in a comparable way as it does for IgG1 (17, 19). Remarkably, the IgG3 point mutant IgG3-E430G performed significantly more efficiently than the respective parental IgG3 on these tumor cell lines, a difference that was not observed when targeted against bacterial polysaccharides (23).

We then quantified IgG oligomerization on antigenic membranes (Fig. 4). The abundance of large IgG oligomers was dependent on the IgG subclass—IgG1 was most effective in forming these, followed by IgG3, IgG2, and IgG4. The introduction of the E430G point mutation increased the abundance of large IgG oligomers and thus multivalent targets for C1 in all subclasses except in IgG3-E430G-DNP which stayed at the level of IgG3-DNP under these conditions. Remarkably, IgG3 hexamers extended up to 15 nm above the lipid membrane, further than the other subclasses (10-11 nm), but less than in a recently proposed structural model of IgG3 hexamers (19 nm), where bivalently bound Fab_2_s were assembled in an array-like arrangement below the Fc platform. HS-AFM imaging of IgG3 hexamers revealed additional, moderately mobile lower structures surrounding the central Fc platform which were absent in case of IgG1 hexamers. We interpret these structures as bivalently antigen-bound IgG3 Fab_2_ (Fig. 3) that are connected to the Fc platform via the long flexible hinge of IgG3, resembling a proposed transition state (18). Maximum elevation is thus likely only reached when antigen mobility (gel phase lipids in this study vs. fluid phase lipids in Ref. (18)) and lateral antigen sizes allow sufficient proximity of individual Fab_2_ within an IgG3 hexamer to establish intermolecular Fab–Fab contacts responsible for array formation.

C1 binding to antigen-bound IgG oligomers was quantified in QCM experiments (Fig. 5) on three different antigen densities. IgG3 was most effective, followed by IgG1, IgG2, and IgG4. IgG3 outperformed IgG1 at low antigen densities, confirming a recent study showing that IgG3 is superior to IgG1 in inducing CDC at low antigen densities (32). IgG4 bound C1 only transiently at medium antigen densities which was not sufficient to lyse DNP-labeled vesicles (Fig. 8). It further did not recruit any C1 at high IgG4 densities (Fig. 5), suggesting that molecular crowding effectively hindered oligomer formation and thus C1 binding supporting the notion of IgG4 acting as a “blocking antibody” (33). Although IgG2 did not induce CDC of tumor cells, the formation of large IgG2 oligomers (Fig. 4), C1 binding (Fig. 5) and complement mediated lysis of DNP-labeled vesicles (Fig. 8) has been observed, albeit with less potency compared to IgG1 and IgG3. We attribute this to the 2 to 10-fold higher antigen surface densities in our model system as compared to the cell surface CD20 densities on DAUDI and RAJI cells used in CDC assays, highlighting the limitation of complement activation by IgG2 to targets expressing elevated antigen densities like bacterial polysaccharides (20, 31). The E430G variants of each IgG subclass approached maximal C1 recruitment efficiency already at lower surface densities than the respective parental IgG, suggesting a more effective oligomerization under these conditions. Accordingly, lysis of DNP-labeled vesicles induced by IgG1-E430G-DNP, IgG2-E430G-DNP, and IgG4-E430G-DNP improved by the introduction of the mutation; however, as observed in the HS-AFM-derived IgG oligomer distributions, IgG3-E430G-DNP was not different from IgG3-DNP.

The E430G point mutation is not part of the Fc–Fc interaction site, instead increasing the flexibility of the IgGs Fc domains by removing a salt bridge that stabilizes the CH2−CH3 interface packing (19, 22). This is notably different to the previously described CDC-enhancing mutation E345R, which directly mutates the Fc–Fc interaction site and results in soluble hexamers (21). Our current model of IgG1 oligomerization (17, 19) suggests that antigen density and affinity, as well as IgG flexibility, are crucial for oligomerization, especially when an antigen-bound IgG recruits an additional IgG from solution via Fc–Fc interactions. In this situation, the E430G point mutation adds to the overall flexibility of the IgG that is otherwise mainly given by the hinge flexibility, increasing the chance to bind a nearby antigen, resulting in a successful oligomerization attempt. This leads to larger IgG oligomers and thus more efficient C1 binding at low antigen densities compared to the respective parental IgGs that lack that additional flexibility. CDC of B cells induced by anti-CD20 IgG1-7D8 ± E430G in a 10-fold range of different CD20 expression levels followed a similar trend (35). IgG3-E430G exhibited slightly improved C1 recruitment with respect to already highly flexible IgG3 only at the lowest antigen density. Length and flexibility of the hinge region vary extensively among IgG subclasses, decreasing in the order IgG3 > IgG1 > IgG4 > IgG2 (6, 7), and resulting in different reach and rotational adaptability that enable them to bivalently bind variably spaced epitopes. Hinge-dependent Fab-Fc flexibility was found to correlate with the complement-activating ability of different IgG subclasses and species. It was suggested that constraints in the upper hinge limit flexibility and leads to steric interference of Fab arms with C1q binding (25). Alternatively, considering the role of hinge flexibility (and the structural impact of the E430G point mutation on the Fc flexibility) in our IgG oligomerization model, we arrive at a similar correlation between IgG flexibility and oligomerization propensity, which in turn correlates with complement recruitment and activation.

Additionally, the relative capacity of the four IgG subclasses to activate complement is associated with their respective monovalent affinities for the gC1q headpieces (4, 5). We found that complement binding and CDC correlate with IgG oligomerization propensities and can be increased by a point mutation that enhances oligomerization but leaves the binding sites for gC1q unaltered (36). To clarify the role of gC1q affinity vs. oligomerization ability of the different IgG subclasses, we developed a mechanistic model of the interactions between C1/C1q and IgG oligomers (*SI Appendix*, Fig. S5) and analyzed respective QCM sensorgrams obtained in the presence of a high-affinity competitor for gC1q (Fig. 6). This allowed us to extract association and dissociation rate constants that govern the kinetics of the interactions. We further introduced a parameter that accounts for the effective concentration (c_eff_) of an unbound gC1q head within an oligomer-bound C1/C1q molecule, which was found to be four times larger in C1 than in C1q, quantifying the contribution of C1r_2_s_2_ proteases to the stability of the IgG hexamer C1 complex (20). The effective arm length of the collagen-like C1q segments resulting from these concentrations (Table 2) agree reasonably well with the respective arm length measured from either the N-terminal stalk region to the gC1q head (C1q) or from the position of the C1r_2_s_2_ heterotrimer to the gC1q head in C1 (2). K_D_ values of the monovalent interactions were ~10 times lower than previously reported, in the order IgG3 < IgG1 < IgG4 < IgG2, which can be explained by the fact that these earlier experiments were performed in solution (5), where IgG conformations differ significantly (37) from the ordered oligomers observed on antigenic membranes (1–3). Experiments involving the unspecific adsorption of IgGs to solid surfaces (38), resulted in comparable K_D_ values as determined here. Although IgG4 did not interact with C1 in this earlier study, its Fc fragment exhibited comparable K_D_ values for gC1q of ~4 to 7 µM as IgG1 Fc fragments. These values are in excellent agreement with the K_D_ values determined here for the interaction of IgG1/IgG4 within hexamers and gC1q suggesting a similarity between the Fc platform of IgG hexamers and individual Fc fragments. While the binding site for gC1q located in the Fc-CH2 domain close to the hinge is likely largely masked by the Fabs within IgGs that float freely in solution (especially when the hinge is short and relatively stiff as in case of IgG4) (25), the fixation of at least one of the Fabs within an IgG hexamer through antigen binding and the orientation of the Fc domain within the Fc platform will likely increase the accessibility of the gC1q binding site, similarly as the enzymatic removal of the Fabs does when Fc fragments are generated from IgGs.

Provided that at least four gC1q heads must bind to adjacent IgGs within an oligomer (17) apply to all four subclasses, the functional affinities of tetramers and larger oligomers are in the pM range and below (Fig. 7 *A* and *B*), so that at physiological C1 concentrations, strong binding and activation should occur as soon as at least tetramers are present on a target cell surface. The monovalent affinity for gC1q heads thus likely plays a rather subordinate role, except in governing the transient unbinding/rebinding rates of gC1q within the complexes, which could affect C1r/C1s activation in the individual subclasses. Removal of C1r_2_s_2_ from IgG-bound C1 by serum concentrations of human C1-ersterase inhibitor (C1-INH) was reported to induce vast dissociation of C1q from IgG2 (20). In our model, removal of C1r_2_s_2_ corresponds to a switch from c_eff_ = 1.5 mM (C1) to 0.4 mM (C1q) leading to a loss in functional affinity that indeed results in a strong dissociation of C1q from IgG2 trimers and tetramers as compared to C1, whereas IgG1 and IgG3 oligomers are less or not at all affected (*SI Appendix*, Fig. S6). Several studies report apparent K_D_ values for C1 binding to antibody-opsonized cells in the 1 to 100 nM range (1, 39). Real-time cell-binding assays detected two distinct binding modes of C1q characterized by K_D_ values of 0.07 to 2.3 and 5 to 70 nM, respectively (40). These values coincide with the K_D_ values determined here (Fig. 8*B*) for the interaction of C1q with IgG1 trimers (0.44 nM) and IgG1 dimers (23.9 nM), which are, however, unable to activate complement (17). Yet, C1 binding levels needed to kill antibody opsonized cells are well below saturating C1 levels. For instance, C3b deposition and CDC are already maximal for anti-CD20 antibody ofatumumab (OFA) -opsonized RAJI cells at less than 10% of its maximum C1 binding level (39), and the E430G point mutant of anti-CD20 IgG1-7D8 induced maximum CDC already at 1 to 2 nM C1, while much higher C1 concentrations were needed to fully saturate C1 binding (28). We found that IgGs exist as oligomer populations on antigenic surfaces with larger oligomers being much less abundant than smaller ones (Fig. 4), and that the functional affinity increases distinctly with oligomer size. IgG1 tetramers and larger (Fig. 7*A*) bind C1 with a sub-pM functional affinity, and, provided that equilibrium is reached (Fig. 7*D* exemplified for an IgG hexamer), binding saturates and induces maximum CDC well below maximum C1 binding levels that are dominated by binding to dimers and trimers. Consequently, maximum C1 binding levels are not a good predictor for complement activation since it is dominated by contributions from non-C1-activating, small IgG oligomers. The respective C1 binding levels reached after incubation generate a C1 concentration dependency for all the large C1-activating IgG oligomers, similar to what was observed in CDC lysis experiments (Fig. 7 *C* and *E*) (28). Lower densities of large IgG1 oligomers in combination with the presence of differing amounts of complement regulatory proteins such as CD46, CD55, and CD59 likely further shift the concentration dependency to higher C1 concentrations, which might explain the differences observed among different antibody variants and cell lines (28). This will be the subject of future investigations.

Under in vivo C1 concentrations, binding of C1 to complement-activating IgG tetramers to hexamers, but also to antigen-bound IgG dimers and trimers (but not to monomers) will saturate, which might be relevant for noncomplement activation-related functions of C1 such as its role as ligand for cell surface receptors on innate and adaptive immune cells (41), or as competitors for Fcγ receptors that exhibit K_D_ values for IgGs in a similar range (42). Taken together, we present comprehensive insights into the complex interplay of innate antibody properties and multivalent IgG–C1q/C1 interactions, that determine IgG subclass–specific differences in complement recruitment and activation. Furthermore, we suggest how complement C1 binding to large oligomers is related to the outcome of CDC, which might offer a way to characterize oligomer distributions and the impact of complement regulatory proteins on different target cells. These insights will allow IgG subclass-specific differences to be specifically adapted and exploited in future immunotherapies.

## Materials and Methods

More details on the materials and methods used in this study are provided in *SI Appendix*, *Materials and Methods*.

### High-Speed Atomic Force Microscopy (HS-AFM).

HS-AFM (43) (SS-NEX, RIBM Ltd., Ibaraki, JP) was conducted in tapping mode at RT (room temperature, 25 °C) in liquid, with typical free amplitudes of 1.5 to 2.5 nm and amplitude setpoints larger than 90%. Silicon nitride cantilevers with electron-beam deposited tips USC-F1.2-k0.15 (NanoWorld® AG, Neuchatel, CH), nominal spring constants of 0.15 N m^−1^, resonance frequencies around 500 kHz, and a quality factor of approx. 2 in liquids were used.

### Quartz Crystal Microbalance (QCM).

QCM experiments were done using a two-channel QCM-I system (MicroVacuum). AT cut SiO2-coated quartz crystals with a diameter of 14.0 mm and a resonance frequency of 5 MHz were used (Quartz Pro AB). All sensorgrams were recorded on the first, third, and fifth harmonic frequencies.

The data shown are related to the third harmonic.

### Data fitting and simulations.

Fitting of dose–response curves to CDC and vesicle-based complement lysis assays, fitting of QCM sensorgrams, and simulation of C1/C1q binding to IgG oligomers according to our mechanistic model (*SI Appendix*, Fig. S5) were done in MATLAB (The MathWorks Inc., MA). For the latter, the ODE15s and fminsearch nonlinear solver were used. CI of fit parameters were determined according to refs. 44, 45.

## Supplementary Material

Appendix 01 (PDF)

Movie S1. HS-AFM movie of two IgG3-DNP hexamers and four IgG3-DNP monomers bound to a DNP-SLB recorded at a scan speed of 2 s/frame. Scan size: 200 x 200 nm^2^ (100 x 100 pixel). Color scale range: 0 - 15 nm.

Movie S2. HS-AFM movie of an IgG3-DNP hexamer, an IgG3-DNP tetramer and IgG3-DNP monomers bound to a DNP-SLB recorded at a scan speed of 2 s/frame. Scan size: 200 x 200 nm^2^ (100 x 100 pixel). Color scale range: 0 - 16 nm.

Movie S3. HS-AFM movie of an IgG1-DNP hexamer and an IgG1-DNP monomer bound to a DNP-SLB recorded at a scan speed of 2 s/frame. Scan size: 200 x 200 nm^2^ (100 x 100 pixel). Color scale range: 0 - 13 nm.

Movie S4. HS-AFM movie of an IgG3-DNP hexamer bound to a DNP-SLB recorded at a scan speed of 2 s/frame. Scan size: 100 x 100 nm^2^ (100 x 100 pixel). Color scale range: 0 - 15 nm.

## Data Availability

All study data are included in the article and/or supporting information.
