# Supplementary material for: Complement activation by IgG subclasses is governed by their ability to oligomerize upon antigen binding
Source: Proc Natl Acad Sci U S A. 2024 Oct 22;121(44):e2406192121. doi: 10.1073/pnas.2406192121 (PMC11536094; doi:10.1073/pnas.2406192121)
Supplement: Supplementary file 1 — Appendix 01 (PDF) [file pnas.2406192121.sapp.pdf]

Supporting Information for

Complement activation by IgG subclasses is governed by their ability to oligomerize upon antigen binding

Nikolaus Frischauf[a,1], Jürgen Strasser[a,1], Ellen G.F. Borg[b], Aran F. Labrijn[b], Frank J. Beurskens[b], Johannes Preiner[a,2]

[a] University of Applied Sciences Upper Austria, Linz, Austria

[b] Genmab, Utrecht, Netherlands

[2]Johannes Preiner

Email: johannes.preiner@fh-linz.at

[1]N.F. and J.S. contributed equally to this work.

This PDF file includes:

Supplementary Material and Methods
Supporting text
Figures S1 to S6
Legends for Movies S1 to S4
SI References

Other supporting materials for this manuscript include the following:

Movies S1 to S4

Supplementary Materials and Methods

DNP labeled liposomes

DNP-labeled liposomes consisting of 1,2-dipalmitoyl-sn-glycero-3-phosphocholine (DPPC), 1,2-dipalmitoyl-sn-glycero-3-phosphoethanolamine (DPPE) and 1,2-dipalmitoyl-sn-glycero-3-phosphoethanolamine-N-[6-[(2,4-dinitrophenyl)amino]hexanoyl] (DNP-cap-DPPE) were used to generate supported lipid bilayers (SLBs) on mica and SiO2 substrates. The lipids were purchased from Avanti Polar Lipids, mixed at different ratios of DPPC:DPPE:DNP-cap-DPPE (90:5:5, 90:9.5:0.5, and 90:9.9:0.1 molar ratios), and dissolved in a 2:1 mixture of chloroform and methanol. After the solvents were rotary-evaporated for 30 min, the lipids were again dissolved in chloroform, which was then rotary-evaporated for 30 min. Drying was completed at a high vacuum pump for 2 h. The lipids were dissolved in 500 µL Milli-Q H2O while immersed in a water bath at 60°C, flooded with argon, and sonicated for 3 min at 60°C to create small unilamellar vesicles. These were diluted to 2 mg/mL in buffer #1 (10 mM HEPES, 150 mM NaCl, 2 mM CaCl2, pH 7.4) and frozen for storage using liquid N2. 0.1%, 0.5%, and 5% DNP-cap-DPPE content of SLBs corresponds to 1.6, 8.1 and 81 x 10^3 DNP molecules/ μm^2.

Supported lipid bilayers and HS-AFM data evaluation

DNP labeled supported lipid bilayers (DNP-SLBs) for HS-AFM were prepared on muscovite mica. The liposomes were incubated on the freshly cleaved surface (0.5 mg/ml in buffer #1), placed in a humidity chamber to prevent evaporation, and heated to 60°C for 30 min. Then the temperature was gradually reduced to RT within 30 min, followed by exchanging the solution with buffer #1. After 10 min of equilibration at RT, and 15 more buffer exchanges, the SLB was ready for imaging. To passivate any exposed mica, SLBs were incubated with 330 nM IgG1-b12 (isotype control antibody against HIV-1 gp120) (44) for 10 min before the molecules of interest were added. The height distributions (Figures 2) were obtained after incubating DNP-SLBs with 33.3 nM of the respective IgG variant for 5 min. The sample was then imaged with a frame size of 400 x 400 nm^2 in buffer #1, and for each distinct location on the sample (n > 15) a short video was recorded (2 - 5 frames). The position of the individual particles and complexes within these videos were tracked using the ImageJ (NIH) plugin Mosaic Suite Particle Tracker (45) and correlated with the respective height information via a MATLAB (The MathWorks Inc., MA, US) script developed in-house. The height average was determined for each particle over the course of the video, pooled for each IgG variant, and plotted as probability density functions (pdfs) and histograms. The oligomer distributions on DNP-SLBs were analyzed in a two-step process: Individual particle dimensions were determined by HS-AFM, and their oligomeric state was further confirmed via their decay pattern determined in subsequent forced dissociation experiments (17). In brief, molecules were scanned in a non-disrupting manner to gauge their number, height, and shape. Subsequently, the scanning force exerted by the HS-AFM cantilever tip is increased (by decreasing the setpoint-amplitude) to dissociate oligomers into their constituent IgGs. Geometric parameters and oligomer decay patterns are combined to assign each IgG assembly its oligomeric state.

Sample preparation and data evaluation in QCM experiments

Before each set of QCM experiments, the SiO_2-coated crystals were cleaned by immersion in 2% sodium dodecyl sulfate (SDS) for 30 min, followed by thorough rinsing with Milli-Q H_2O. The chips were dried in a gentle stream of N_2 and oxidized using air plasma (4 min at 80 W; Diener electronic GmbH & Co. KG, Ebhausen, DE), then mounted in the measurement chamber. The sensor surface and the fluid system was further cleaned by a flow of 2% SDS at 250 µL min^{-1} for 5 min, followed by Milli-Q H_2O at 250 µL min^{-1} for 5 min directly before the measurements. Before lipid incubation, the flow was stopped and the measurement chamber was heated up to 45°C, left on this temperature for a few minutes and cooled down again to 22°C for equilibration. To generate DNP-SLBs on QCM chips, the DPPC:DPPE:DNP-cap-DPPE liposome stock solution was heated to 60°C for 30 min and then slowly cooled to RT within 30 min. The solution was ready for injection after dilution to 200 µg/mL with buffer #1. DNP-SLB formation was typically complete after 30 min at µL min^{-1}, after which the flow medium was changed to buffer #1 and a second heat cycle was started, followed by equilibration in buffer #1. Finally, a control injection with 15.2 nM C1q (Complement Technology Inc., TX, US), was performed to check for imperfections in the DNP-SLBs. Competition

experiments (Fig. 6) were performed in either buffer #1 (C1q) or buffer #2 (155 mM NaCl, 9.6 mM HEPES, 1.9 mM $CaCl_2$, 1.8 mM sodium acetate, 1.8 mM EACA, 0.4 mM benzamidin HCl, 0.4 mM EDTA, 1.4% glycerol, pH 7.4; C1).
Raw-sensorgrams (in Hz *vs.* time) were converted to molecule densities on the QCM chip (molecules/µm^2 *vs.* time) by determining the bound mass according to the Sauerbrey equation (46) which relates the change in resonance frequency ∆f of a quartz crystal oscillating in thickness shear mode to the mass adsorbed on its surface ∆m by the relation $\Delta f = \frac{2f_0^2}{\sqrt{\rho_q \mu_q}} \cdot \frac{\Delta m}{A}$. Here f_0 is the fundamental resonance frequency, and ρ_q and μ_q are the crystal density (2.648 g cm^{-3}) and its shear modulus (2.947 10^{11} g cm^{-1} s^2), respectively. The resulting masses were corrected for the effect of trapped water according to Höök *et al.* (47). Determination of molecule densities further requires the effective surface area of the DNP-SLBs, which may exceed the actual working surface area of the QCM chip due to residual, only partially fused (and thus non-flat) vesicles. We corrected the active surface area accordingly by comparing the theoretical mass of a flat DNP-SLB covering the working surface (average molecular weight of 729.3 Da for DPPC:DPPE=9:1; head-group area of 0.626 nm^2 (48); 0.5 nm buffer layer between SiO_2 and DNP-SLB) to the observed mass, and modeling the excess as buffer filled half spheres with a diameter ~ 35 nm (determined by HS-AFM imaging).

Cell lines

FreeStyle™ Expi293F™ cells were cultured in FreeStyle™ 293 expression medium according to the manufacturer's instructions (Invitrogen). Additional cell lines were obtained from the American Type Culture Collection (ATCC). Raji and Daudi (human CD20-positive Burkitt's lymphoma) cells were cultured in RPMI 1640 medium (Lonza), supplemented with 10% (v/v) heat-inactivated Donor Bovine Serum with Iron (DBSI; Life Technologies). Wien-133 (human CD52-positive Burkitt's lymphoma) cells were cultured in Iscove's Modified Dulbeco's Medium (IMDM) with HEPES and L-Glutamine (Lonza), supplemented with 10% (v/v) heat-inactivated DBSI. All cell lines were maintained at 37°C in a 5% (v/v) CO_2 humidified incubator. Based on a diameter of 5 - 8 µm (12) and respective CD20 expression levels per cell (1) the CD20 surface densities are estimated to be 1.4 – 3.6 x 10^3 CD20 molecules/µm^2 on DAUDI cells and 0.7 – 1.7 x 10^3 CD20 molecules/µm^2 on RAJI and WIEN cells.

Construction, expression, and purification of antibody variants

Antibody heavy-chain (HC) expression vectors were constructed by inserting de novo synthesized (Geneart) codon optimized HC coding regions into expression vector pcDNA3.3 (Invitrogen). The HC coding regions consisted of the VH regions of mAbs 7D8 (human CD20-specific (2)), CAMPATH (human CD52-specific (3)), G2a2 (DNP-specific (4)) or b12 (HIV-1 gp120-specific (5)), genetically fused to the CH regions of wild-type human IgG1*03, IgG2*01, IgG3*01 or IgG4*01 or one of the mutants variants containing the E430G point mutation (1) or the RGY (E345R-E430G-S440Y) triple-mutation (6) (EU numbering conventions are used throughout the manuscript). Likewise, separate light-chain expression vectors were constructed by inserting the corresponding VL coding regions in frame with the CL coding regions of the human (J00241) kappa light chain into expression vector pcDNA3.3.
All antibodies were produced under serum-free conditions by co-transfecting relevant heavy and light chain expression vectors in FreeStyle™ Expi293F™ cells, using ExpiFectamine™ 293 (LifeTechnologies), according to the manufacturer's instructions. IgG1, IgG2 and IgG4 antibody variants were purified by protein A affinity chromatography (MabSelect SuRe; GE Health Care), dialyzed overnight to PBS and filter-sterilized over 0.2-µM dead-end filters. Alternatively, IgG3 antibody variants were purified by protein G affinity chromatography (GE Health Care). Purity was determined by CE-SDS and concentration was measured by absorbance at 280 nm (specific extinction coefficients were calculated for each protein). Batches of purified antibody were tested by high-performance size-exclusion chromatography (HP-SEC) for aggregates or degradation products and shown to be at least 95% monomeric. Purified antibodies were stored at 2 - 8°C.

Complement dependent cytotoxicity (CDC) assay

The capacity of anti-CD20 and anti-CD52 antibodies to induce CDC was assessed by pre-incubating Raji ($1x10^5$ cells), Daudi ($1x10^5$ cells) or Wien133 target cells ($3x10^5$ cells) in assay buffer (RPMI medium containing 0.1% (w/v) BSA) at 21°C for 15 min with serial diluted antibodies. Pooled human serum (20% (v/v)) was added as a source of complement and cells were incubated at 37°C for an additional 45 min. Cells were then put on ice and viability was determined by staining with propidium iodide (PI) and detected using an iQue screener (Intellicyt). Percentage lysis was calculated using the following formula: % lysis = (experimental release (fluorescence) – spontaneous release without antibody (fluorescence))/ (maximal release of IgG1 (fluorescence) – spontaneous release without antibody (fluorescence)) x 100.

DNP-labeled liposomal vesicle-based complement lysis assay

For the DNP-labeled liposomal vesicle-based complement lysis assay we used the Wako Auto Kit (Wako Pure Chemical, Chuo-Ku Osaka, Japan) with an adapted protocol to be able to test the anti-DNP variants used. In this assay, the enzyme glucose-6-phosphate dehydrogenase (G6PDH) is entrapped within the vesicles, and the substrate beta-Nicotinamide adenine dinucleotide (NAD^+) is contained in the surrounding buffer. As soon as the vesicles become leaky as a result of MAC formation, G6PDH can react with NAD^+ converting it into NADH. The rate of NADH production is measured in terms of absorbance per time, which is proportional to the available enzyme concentration that is no longer entrapped in vesicles which in turn is directly proportional to the concentration of leaky vesicles (7).

In detail, in a regular 96 ELISA flat bottom plate, 15% normal human serum (Sanquin, Amsterdam, The Netherlands) was added to the liposome mixture (R1 from the kit) containing G6PDH and a serial dilution of antibody (20 x concentrated) and incubated for 5 min at room temperature. Then 33.5% custom substrate mix (containing 24 mM D-glucose-6-phospate solution, 9 mM NAD, 20.6 mM lactose monohydrate, 9.8 mM NaOH (Sigma), 1:1 mixed with maleate buffer (R2a from the kit)) was added and incubated at room temperature and measured kinetically on an Envision microplate reader (PerkinElmer, Waltham, MA) at 340 nm for 20 min. The change in absorbance (Abs per second was calculated from the linear part of the curve) dAbs/dt was plotted against log transformed antibody concentration.

Supporting Information Text

Mechanistic model of C1/C1q binding to IgG oligomers

We have deduced the system of rate equations governing the time course of C1q/C1 binding/dissociation to/from different IgG oligomers from the kinetic scheme given in Fig. S5. The model is based on the assumption that after the removal of IgG from solution, the resulting IgG oligomer distributions do not change over time, which is justified by the observation that after dissociation of C1q/C1 induced by a competitor, the subsequent C1q/C1 incubations on the very same IgG-opsonized DNP-SLBs led to similarly shaped sensorgrams and reached similar binding levels as the respective preceding experiments. For the different states of IgG-oligomer – C1q/C1 – competitor complexes we introduced a three-digit index notation y_{nmo}, where *n, m, and o* depict the size of the IgG oligomer, the number of available (i.e. not yet bound to an IgG or a competitor) gC1q heads, and the number of competitor-bound gC1q heads within a C1q/C1 molecule, respectively. For example, y_{512} (Fig. S5B, 3rd row, 3rd column) represents a C1 molecule bound to an IgG pentamer (5), having one gC1q head still unbound and thus available (1), and two gC1q heads occupied by a competitor (2), and consequently 6-1-2 = 3 gC1q heads already bound to the IgG pentamer. The corresponding rate equation that governs the time evolution of this state can be obtained by adding up all flux from neighboring states into/from y_{512}. Starting from left to right and from bottom to top we get:

$$\frac{d\boldsymbol{y}_{512}}{dt} = \left(2 \cdot \boldsymbol{k}_{on,comp}\boldsymbol{C}_C\boldsymbol{y}_{521} - 2 \cdot \boldsymbol{k}_{off,comp}\boldsymbol{y}_{512}\right) - \left(1 \cdot \boldsymbol{k}_{on,comp}\boldsymbol{C}_C\boldsymbol{y}_{512} - 3 \cdot \boldsymbol{k}_{off,comp}\boldsymbol{y}_{503}\right) - \left(2 \cdot \frac{1}{3} \cdot \boldsymbol{k}_{on}\boldsymbol{c}_{eff}\boldsymbol{y}_{512} - 4 \cdot \boldsymbol{k}_{off}\boldsymbol{y}_{502}\right) + \left(3 \cdot \frac{2}{4} \cdot \boldsymbol{k}_{on}\boldsymbol{c}_{eff}\boldsymbol{y}_{522} - 3 \cdot \boldsymbol{k}_{off}\boldsymbol{y}_{512}\right)$$

The numerical pre-factors of the respective expressions account for the respective probabilities that a certain transition may occur, by assuming that only adjacent gC1q heads may bind to adjacent IgGs within an oligomer. IgG oligomers without C1/C1q bound are depicted by single-digit indexes, e.g. y_5 for an IgG pentamer.

Notably, the cryo-ET structures of soluble C1-IgG1 hexamer complexes reported in Ref. (8) were categorized as separate classes with four, five, or six gC1q domains in contact with Fc platforms. The class with six gC1q heads bound appeared most frequently, followed by four and five gC1q domains bound, suggesting that C1 bound with an even number of gC1q heads are more stable (per bond) than the ones bound with an odd number. In contrast, our model does not differentiate between even and odd numbers of bound gC1q heads and thus predicts a monotonic increase of the number of gC1q heads bound to the Fc platform within an ensemble of C1-IgG1 hexamer complexes. Introduction of an additional parameter accounting for a pair-wise cooperativity in gC1q binding (practically by introducing two different c_{eff} that apply alternatingly) would in principle enable such a non-monotonic behavior as observed in cryo-ET structures, however, given that our model already reasonably fits our QCM data, and only marginal improvements could be expected from a more complex model, we retained the simpler model.

The complete set of rate equations then reads (next page):

I. **C1/C1q binding to IgG hexamers (Fig. S5A):**

1st row (one gC1q head bound):

$$\frac{d\boldsymbol{y}_{650}}{dt} = -\left(5\cdot\boldsymbol{k}_{on,comp}\boldsymbol{C}_C\boldsymbol{y}_{650} - 1\cdot\boldsymbol{k}_{off,comp}\boldsymbol{y}_{641}\right) - \left(5\cdot\boldsymbol{k}_{on}\boldsymbol{c}_{eff}\boldsymbol{y}_{650} - 2\cdot\boldsymbol{k}_{off}\boldsymbol{y}_{640}\right) + \left(36\cdot\boldsymbol{k}_{on}\boldsymbol{C}_{C1}\boldsymbol{y}_{6} - \boldsymbol{k}_{off}\boldsymbol{y}_{650}\right)$$

$$\frac{d\boldsymbol{y}_{641}}{dt} = \left(5\cdot\boldsymbol{k}_{on,comp}\boldsymbol{C}_C\boldsymbol{y}_{650} - 1\cdot\boldsymbol{k}_{off,comp}\boldsymbol{y}_{641}\right) - \left(4\cdot\boldsymbol{k}_{on,comp}\boldsymbol{C}_C\boldsymbol{y}_{641} - 2\cdot\boldsymbol{k}_{off,comp}\boldsymbol{y}_{632}\right) - \left(4\cdot\boldsymbol{k}_{on}\boldsymbol{c}_{eff}\boldsymbol{y}_{641} - 2\cdot\boldsymbol{k}_{off}\boldsymbol{y}_{631}\right) + \left(-\boldsymbol{k}_{off}\boldsymbol{y}_{641}\right)$$

$$\frac{d\boldsymbol{y}_{632}}{dt} = \left(4\cdot\boldsymbol{k}_{on,comp}\boldsymbol{C}_C\boldsymbol{y}_{641} - 2\cdot\boldsymbol{k}_{off,comp}\boldsymbol{y}_{632}\right) - \left(3\cdot\boldsymbol{k}_{on,comp}\boldsymbol{C}_C\boldsymbol{y}_{632} - 3\cdot\boldsymbol{k}_{off,comp}\boldsymbol{y}_{623}\right) - \left(3\cdot\boldsymbol{k}_{on}\boldsymbol{c}_{eff}\boldsymbol{y}_{632} - 2\cdot\boldsymbol{k}_{off}\boldsymbol{y}_{622}\right) + \left(-\boldsymbol{k}_{off}\boldsymbol{y}_{632}\right)$$

$$\frac{d\boldsymbol{y}_{623}}{dt} = \left(3\cdot\boldsymbol{k}_{on,comp}\boldsymbol{C}_C\boldsymbol{y}_{632} - 3\cdot\boldsymbol{k}_{off,comp}\boldsymbol{y}_{623}\right) - \left(2\cdot\boldsymbol{k}_{on,comp}\boldsymbol{C}_C\boldsymbol{y}_{623} - 4\cdot\boldsymbol{k}_{off,comp}\boldsymbol{y}_{614}\right) - \left(2\cdot\boldsymbol{k}_{on}\boldsymbol{c}_{eff}\boldsymbol{y}_{623} - 2\cdot\boldsymbol{k}_{off}\boldsymbol{y}_{613}\right) + \left(-\boldsymbol{k}_{off}\boldsymbol{y}_{623}\right)$$

$$\frac{d\boldsymbol{y}_{614}}{dt} = \left(2\cdot\boldsymbol{k}_{on,comp}\boldsymbol{C}_C\boldsymbol{y}_{623} - 4\cdot\boldsymbol{k}_{off,comp}\boldsymbol{y}_{614}\right) - \left(1\cdot\boldsymbol{k}_{on,comp}\boldsymbol{C}_C\boldsymbol{y}_{614} - 5\cdot\boldsymbol{k}_{off,comp}\boldsymbol{y}_{605}\right) - \left(1\cdot\boldsymbol{k}_{on}\boldsymbol{c}_{eff}\boldsymbol{y}_{614} - 2\cdot\boldsymbol{k}_{off}\boldsymbol{y}_{604}\right) + \left(-\boldsymbol{k}_{off}\boldsymbol{y}_{614}\right)$$

$$\frac{d\boldsymbol{y}_{605}}{dt} = \left(1\cdot\boldsymbol{k}_{on,comp}\boldsymbol{C}_C\boldsymbol{y}_{614} - 5\cdot\boldsymbol{k}_{off,comp}\boldsymbol{y}_{605}\right) + \left(-\boldsymbol{k}_{off}\boldsymbol{y}_{605}\right)$$

2nd row (two gC1q heads bound):

$$\frac{d\boldsymbol{y}_{640}}{dt} = -\left(4\cdot\boldsymbol{k}_{on,comp}\boldsymbol{C}_C\boldsymbol{y}_{640} - 1\cdot\boldsymbol{k}_{off,comp}\boldsymbol{y}_{631}\right) - \left(4\cdot\boldsymbol{k}_{on}\boldsymbol{c}_{eff}\boldsymbol{y}_{640} - 3\cdot\boldsymbol{k}_{off}\boldsymbol{y}_{630}\right) + \left(5\cdot\boldsymbol{k}_{on}\boldsymbol{c}_{eff}\boldsymbol{y}_{650} - 2\cdot\boldsymbol{k}_{off}\boldsymbol{y}_{640}\right)$$

$$\frac{d\boldsymbol{y}_{631}}{dt} = \left(4\cdot\boldsymbol{k}_{on,comp}\boldsymbol{C}_C\boldsymbol{y}_{640} - 1\cdot\boldsymbol{k}_{off,comp}\boldsymbol{y}_{631}\right) - \left(3\cdot\boldsymbol{k}_{on,comp}\boldsymbol{C}_C\boldsymbol{y}_{631} - 2\cdot\boldsymbol{k}_{off,comp}\boldsymbol{y}_{622}\right) - \left(3\cdot\boldsymbol{k}_{on}\boldsymbol{c}_{eff}\boldsymbol{y}_{631} - 3\cdot\boldsymbol{k}_{off}\boldsymbol{y}_{621}\right) + \left(4\cdot\boldsymbol{k}_{on}\boldsymbol{c}_{eff}\boldsymbol{y}_{641} - 2\cdot\boldsymbol{k}_{off}\boldsymbol{y}_{631}\right)$$

$$\frac{d\boldsymbol{y}_{622}}{dt} = \left(3\cdot\boldsymbol{k}_{on,comp}\boldsymbol{C}_C\boldsymbol{y}_{631} - 2\cdot\boldsymbol{k}_{off,comp}\boldsymbol{y}_{622}\right) - \left(2\cdot\boldsymbol{k}_{on,comp}\boldsymbol{C}_C\boldsymbol{y}_{622} - 3\cdot\boldsymbol{k}_{off,comp}\boldsymbol{y}_{613}\right) - \left(2\cdot\boldsymbol{k}_{on}\boldsymbol{c}_{eff}\boldsymbol{y}_{622} - 3\cdot\boldsymbol{k}_{off}\boldsymbol{y}_{612}\right) + \left(3\cdot\boldsymbol{k}_{on}\boldsymbol{c}_{eff}\boldsymbol{y}_{632} - 2\cdot\boldsymbol{k}_{off}\boldsymbol{y}_{622}\right)$$

$$\frac{d\boldsymbol{y}_{613}}{dt} = \left(2\cdot\boldsymbol{k}_{on,comp}\boldsymbol{C}_C\boldsymbol{y}_{622} - 3\cdot\boldsymbol{k}_{off,comp}\boldsymbol{y}_{613}\right) - \left(1\cdot\boldsymbol{k}_{on,comp}\boldsymbol{C}_C\boldsymbol{y}_{613} - 4\cdot\boldsymbol{k}_{off,comp}\boldsymbol{y}_{604}\right) - \left(1\cdot\boldsymbol{k}_{on}\boldsymbol{c}_{eff}\boldsymbol{y}_{613} - 3\cdot\boldsymbol{k}_{off}\boldsymbol{y}_{603}\right) + \left(2\cdot\boldsymbol{k}_{on}\boldsymbol{c}_{eff}\boldsymbol{y}_{623} - 2\cdot\boldsymbol{k}_{off}\boldsymbol{y}_{613}\right)$$

$$\frac{d\boldsymbol{y}_{604}}{dt} = \left(1\cdot\boldsymbol{k}_{on,comp}\boldsymbol{C}_C\boldsymbol{y}_{613} - 4\cdot\boldsymbol{k}_{off,comp}\boldsymbol{y}_{604}\right) + \left(1\cdot\boldsymbol{k}_{on}\boldsymbol{c}_{eff}\boldsymbol{y}_{614} - 2\cdot\boldsymbol{k}_{off}\boldsymbol{y}_{604}\right)$$

3rd row (three gC1q heads bound):

$$\frac{d\boldsymbol{y}_{630}}{dt} = -\left(3\cdot\boldsymbol{k}_{on,comp}\boldsymbol{C}_C\boldsymbol{y}_{630} - 1\cdot\boldsymbol{k}_{off,comp}\boldsymbol{y}_{621}\right) - \left(3\cdot\boldsymbol{k}_{on}\boldsymbol{c}_{eff}\boldsymbol{y}_{630} - 4\cdot\boldsymbol{k}_{off}\boldsymbol{y}_{620}\right) + \left(4\cdot\boldsymbol{k}_{on}\boldsymbol{c}_{eff}\boldsymbol{y}_{640} - 3\cdot\boldsymbol{k}_{off}\boldsymbol{y}_{630}\right)$$

$$\frac{d\boldsymbol{y}_{621}}{dt} = \left(3\cdot\boldsymbol{k}_{on,comp}\boldsymbol{C}_C\boldsymbol{y}_{630} - 1\cdot\boldsymbol{k}_{off,comp}\boldsymbol{y}_{621}\right) - \left(2\cdot\boldsymbol{k}_{on,comp}\boldsymbol{C}_C\boldsymbol{y}_{621} - 2\cdot\boldsymbol{k}_{off,comp}\boldsymbol{y}_{612}\right) - \left(2\cdot\boldsymbol{k}_{on}\boldsymbol{c}_{eff}\boldsymbol{y}_{621} - 4\cdot\boldsymbol{k}_{off}\boldsymbol{y}_{611}\right) + \left(3\cdot\boldsymbol{k}_{on}\boldsymbol{c}_{eff}\boldsymbol{y}_{631} - 3\cdot\boldsymbol{k}_{off}\boldsymbol{y}_{621}\right)$$

$$\frac{d\boldsymbol{y}_{612}}{dt} = \left(2\cdot\boldsymbol{k}_{on,comp}\boldsymbol{C}_C\boldsymbol{y}_{621} - 2\cdot\boldsymbol{k}_{off,comp}\boldsymbol{y}_{612}\right) - \left(1\cdot\boldsymbol{k}_{on,comp}\boldsymbol{C}_C\boldsymbol{y}_{612} - 3\cdot\boldsymbol{k}_{off,comp}\boldsymbol{y}_{603}\right) - \left(1\cdot\boldsymbol{k}_{on}\boldsymbol{c}_{eff}\boldsymbol{y}_{612} - 4\cdot\boldsymbol{k}_{off}\boldsymbol{y}_{602}\right) + \left(2\cdot\boldsymbol{k}_{on}\boldsymbol{c}_{eff}\boldsymbol{y}_{622} - 3\cdot\boldsymbol{k}_{off}\boldsymbol{y}_{612}\right)$$

$$\frac{d\boldsymbol{y}_{603}}{dt} = \left(1\cdot\boldsymbol{k}_{on,comp}\boldsymbol{C}_C\boldsymbol{y}_{612} - 3\cdot\boldsymbol{k}_{off,comp}\boldsymbol{y}_{603}\right) + \left(1\cdot\boldsymbol{k}_{on}\boldsymbol{c}_{eff}\boldsymbol{y}_{613} - 3\cdot\boldsymbol{k}_{off}\boldsymbol{y}_{603}\right)$$

4th row (four gC1q heads bound):

$$\frac{d\boldsymbol{y}_{620}}{dt} = -\left(2\cdot \boldsymbol{k}_{on,comp}\boldsymbol{C}_C\boldsymbol{y}_{620} - 1\cdot \boldsymbol{k}_{off,comp}\boldsymbol{y}_{611}\right) - \left(2\cdot \boldsymbol{k}_{on}\boldsymbol{c}_{eff}\boldsymbol{y}_{620} - 5\cdot \boldsymbol{k}_{off}\boldsymbol{y}_{610}\right) + \left(3\cdot \boldsymbol{k}_{on}\boldsymbol{c}_{eff}\boldsymbol{y}_{630} - 4\cdot \boldsymbol{k}_{off}\boldsymbol{y}_{620}\right)$$

$$\frac{d\boldsymbol{y}_{611}}{dt} = \left(2\cdot \boldsymbol{k}_{on,comp}\boldsymbol{C}_C\boldsymbol{y}_{620} - 1\cdot \boldsymbol{k}_{off,comp}\boldsymbol{y}_{611}\right) - \left(1\cdot \boldsymbol{k}_{on,comp}\boldsymbol{C}_C\boldsymbol{y}_{611} - 2\cdot \boldsymbol{k}_{off,comp}\boldsymbol{y}_{602}\right) - \left(1\cdot \boldsymbol{k}_{on}\boldsymbol{c}_{eff}\boldsymbol{y}_{611} - 5\cdot \boldsymbol{k}_{off}\boldsymbol{y}_{601}\right) + \left(2\cdot \boldsymbol{k}_{on}\boldsymbol{c}_{eff}\boldsymbol{y}_{621} - 4\cdot \boldsymbol{k}_{off}\boldsymbol{y}_{611}\right)$$

$$\frac{d\boldsymbol{y}_{602}}{dt} = \left(1\cdot \boldsymbol{k}_{on,comp}\boldsymbol{C}_C\boldsymbol{y}_{611} - 2\cdot \boldsymbol{k}_{off,comp}\boldsymbol{y}_{602}\right) + \left(1\cdot \boldsymbol{k}_{on}\boldsymbol{c}_{eff}\boldsymbol{y}_{612} - 4\cdot \boldsymbol{k}_{off}\boldsymbol{y}_{602}\right)$$

5th row (five gC1q heads bound):

$$\frac{d\boldsymbol{y}_{610}}{dt} = -\left(1\cdot \boldsymbol{k}_{on,comp}\boldsymbol{C}_C\boldsymbol{y}_{610} - 1\cdot \boldsymbol{k}_{off,comp}\boldsymbol{y}_{601}\right) - \left(1\cdot \boldsymbol{k}_{on}\boldsymbol{c}_{eff}\boldsymbol{y}_{610} - 6\cdot \boldsymbol{k}_{off}\boldsymbol{y}_{600}\right) + \left(2\cdot \boldsymbol{k}_{on}\boldsymbol{c}_{eff}\boldsymbol{y}_{620} - 5\cdot \boldsymbol{k}_{off}\boldsymbol{y}_{610}\right)$$

$$\frac{d\boldsymbol{y}_{601}}{dt} = \left(1\cdot \boldsymbol{k}_{on,comp}\boldsymbol{C}_C\boldsymbol{y}_{610} - 1\cdot \boldsymbol{k}_{off,comp}\boldsymbol{y}_{601}\right) + \left(1\cdot \boldsymbol{k}_{on}\boldsymbol{c}_{eff}\boldsymbol{y}_{611} - 5\cdot \boldsymbol{k}_{off}\boldsymbol{y}_{601}\right)$$

6th row (six gC1q heads bound):

$$\frac{d\boldsymbol{y}_{600}}{dt} = +\left(1\cdot \boldsymbol{k}_{on}\boldsymbol{c}_{eff}\boldsymbol{y}_{610} - 6\cdot \boldsymbol{k}_{off}\boldsymbol{y}_{600}\right)$$

Hexamers without C1/C1q:

$$\frac{d\boldsymbol{y}_{6}}{dt} = -36\cdot \boldsymbol{k}_{on}\boldsymbol{C}_{C1}\boldsymbol{y}_6 + \boldsymbol{k}_{off}(\boldsymbol{y}_{650} + \boldsymbol{y}_{641} + \boldsymbol{y}_{632} + \boldsymbol{y}_{623} + \boldsymbol{y}_{614} + \boldsymbol{y}_{605})$$

II. C1/C1q binding to IgG pentamers (Fig. S5B):

1st row (one gC1q head bound):

$$\frac{d\boldsymbol{y}_{550}}{dt} = -\left(5\cdot \boldsymbol{k}_{on,comp}\boldsymbol{C}_C\boldsymbol{y}_{550} - 1\cdot \boldsymbol{k}_{off,comp}\boldsymbol{y}_{541}\right) - \left(4\cdot \frac{5}{5}\cdot \boldsymbol{k}_{on}\boldsymbol{c}_{eff}\boldsymbol{y}_{550} - 2\cdot \boldsymbol{k}_{off}\boldsymbol{y}_{540}\right) + (30\cdot \boldsymbol{k}_{on}\boldsymbol{C}_{C1}\boldsymbol{y}_5 - \boldsymbol{k}_{off}\boldsymbol{y}_{550})$$

$$\frac{d\boldsymbol{y}_{541}}{dt} = \left(5\cdot \boldsymbol{k}_{on,comp}\boldsymbol{C}_C\boldsymbol{y}_{550} - 1\cdot \boldsymbol{k}_{off,comp}\boldsymbol{y}_{541}\right) - \left(4\cdot \boldsymbol{k}_{on,comp}\boldsymbol{C}_C\boldsymbol{y}_{541} - 2\cdot \boldsymbol{k}_{off,comp}\boldsymbol{y}_{532}\right) - \left(4\cdot \frac{4}{5}\cdot \boldsymbol{k}_{on}\boldsymbol{c}_{eff}\boldsymbol{y}_{541} - 2\cdot \boldsymbol{k}_{off}\boldsymbol{y}_{531}\right) + (\quad -\boldsymbol{k}_{off}\boldsymbol{y}_{541})$$

$$\frac{d\boldsymbol{y}_{532}}{dt} = \left(4\cdot \boldsymbol{k}_{on,comp}\boldsymbol{C}_C\boldsymbol{y}_{541} - 2\cdot \boldsymbol{k}_{off,comp}\boldsymbol{y}_{532}\right) - \left(3\cdot \boldsymbol{k}_{on,comp}\boldsymbol{C}_C\boldsymbol{y}_{532} - 3\cdot \boldsymbol{k}_{off,comp}\boldsymbol{y}_{523}\right) - \left(4\cdot \frac{3}{5}\cdot \boldsymbol{k}_{on}\boldsymbol{c}_{eff}\boldsymbol{y}_{532} - 2\cdot \boldsymbol{k}_{off}\boldsymbol{y}_{522}\right) + (\quad -\boldsymbol{k}_{off}\boldsymbol{y}_{532})$$

$$\frac{d\boldsymbol{y}_{523}}{dt} = \left(3\cdot \boldsymbol{k}_{on,comp}\boldsymbol{C}_C\boldsymbol{y}_{532} - 3\cdot \boldsymbol{k}_{off,comp}\boldsymbol{y}_{523}\right) - \left(2\cdot \boldsymbol{k}_{on,comp}\boldsymbol{C}_C\boldsymbol{y}_{523} - 4\cdot \boldsymbol{k}_{off,comp}\boldsymbol{y}_{514}\right) - \left(4\cdot \frac{2}{5}\cdot \boldsymbol{k}_{on}\boldsymbol{c}_{eff}\boldsymbol{y}_{523} - 2\cdot \boldsymbol{k}_{off}\boldsymbol{y}_{513}\right) + (\quad -\boldsymbol{k}_{off}\boldsymbol{y}_{523})$$

$$\frac{d\boldsymbol{y}_{514}}{dt} = \left(2\cdot \boldsymbol{k}_{on,comp}\boldsymbol{C}_C\boldsymbol{y}_{523} - 4\cdot \boldsymbol{k}_{off,comp}\boldsymbol{y}_{514}\right) - \left(1\cdot \boldsymbol{k}_{on,comp}\boldsymbol{C}_C\boldsymbol{y}_{514} - 5\cdot \boldsymbol{k}_{off,comp}\boldsymbol{y}_{505}\right) - \left(4\cdot \frac{1}{5}\cdot \boldsymbol{k}_{on}\boldsymbol{c}_{eff}\boldsymbol{y}_{514} - 2\cdot \boldsymbol{k}_{off}\boldsymbol{y}_{504}\right) + (\quad -\boldsymbol{k}_{off}\boldsymbol{y}_{514})$$

$$\frac{d\boldsymbol{y}_{505}}{dt} = \left(1\cdot \boldsymbol{k}_{on,comp}\boldsymbol{C}_C\boldsymbol{y}_{514} - 5\cdot \boldsymbol{k}_{off,comp}\boldsymbol{y}_{505}\right) + (\quad -\boldsymbol{k}_{off}\boldsymbol{y}_{505})$$

2nd row (two gC1q heads bound):

$$\frac{d\boldsymbol{y}_{540}}{dt} = -\left(4\cdot \boldsymbol{k}_{on,comp}\boldsymbol{C}_C\boldsymbol{y}_{540} - 1\cdot \boldsymbol{k}_{off,comp}\boldsymbol{y}_{531}\right) - \left(3\cdot \frac{4}{4}\cdot \boldsymbol{k}_{on}\boldsymbol{c}_{eff}\boldsymbol{y}_{540} - 3\cdot \boldsymbol{k}_{off}\boldsymbol{y}_{530}\right) + \left(4\cdot \frac{5}{5}\cdot \boldsymbol{k}_{on}\boldsymbol{c}_{eff}\boldsymbol{y}_{550} - 2\cdot \boldsymbol{k}_{off}\boldsymbol{y}_{540}\right)$$

$$\frac{d\boldsymbol{y}_{531}}{dt} = \left(4 \cdot \boldsymbol{k}_{on,comp}\boldsymbol{C}_C\boldsymbol{y}_{540} - 1 \cdot \boldsymbol{k}_{off,comp}\boldsymbol{y}_{531}\right) - \left(3 \cdot \boldsymbol{k}_{on,comp}\boldsymbol{C}_C\boldsymbol{y}_{531} - 2 \cdot \boldsymbol{k}_{off,comp}\boldsymbol{y}_{522}\right) - \left(3 \cdot \frac{3}{4} \cdot \boldsymbol{k}_{on}\boldsymbol{c}_{eff}\boldsymbol{y}_{531} - 3 \cdot \boldsymbol{k}_{off}\boldsymbol{y}_{521}\right) + \left(4 \cdot \frac{4}{5} \cdot \boldsymbol{k}_{on}\boldsymbol{c}_{eff}\boldsymbol{y}_{541} - 2 \cdot \boldsymbol{k}_{off}\boldsymbol{y}_{531}\right)$$

$$\frac{d\boldsymbol{y}_{522}}{dt} = \left(3 \cdot \boldsymbol{k}_{on,comp}\boldsymbol{C}_C\boldsymbol{y}_{531} - 2 \cdot \boldsymbol{k}_{off,comp}\boldsymbol{y}_{522}\right) - \left(2 \cdot \boldsymbol{k}_{on,comp}\boldsymbol{C}_C\boldsymbol{y}_{522} - 3 \cdot \boldsymbol{k}_{off,comp}\boldsymbol{y}_{513}\right) - \left(3 \cdot \frac{2}{4} \cdot \boldsymbol{k}_{on}\boldsymbol{c}_{eff}\boldsymbol{y}_{522} - 3 \cdot \boldsymbol{k}_{off}\boldsymbol{y}_{512}\right) + \left(4 \cdot \frac{3}{5} \cdot \boldsymbol{k}_{on}\boldsymbol{c}_{eff}\boldsymbol{y}_{532} - 2 \cdot \boldsymbol{k}_{off}\boldsymbol{y}_{522}\right)$$

$$\frac{d\boldsymbol{y}_{513}}{dt} = \left(2 \cdot \boldsymbol{k}_{on,comp}\boldsymbol{C}_C\boldsymbol{y}_{522} - 3 \cdot \boldsymbol{k}_{off,comp}\boldsymbol{y}_{513}\right) - \left(1 \cdot \boldsymbol{k}_{on,comp}\boldsymbol{C}_C\boldsymbol{y}_{513} - 4 \cdot \boldsymbol{k}_{off,comp}\boldsymbol{y}_{504}\right) - \left(3 \cdot \frac{1}{4} \cdot \boldsymbol{k}_{on}\boldsymbol{c}_{eff}\boldsymbol{y}_{513} - 3 \cdot \boldsymbol{k}_{off}\boldsymbol{y}_{503}\right) + \left(4 \cdot \frac{2}{5} \cdot \boldsymbol{k}_{on}\boldsymbol{c}_{eff}\boldsymbol{y}_{523} - 2 \cdot \boldsymbol{k}_{off}\boldsymbol{y}_{513}\right)$$

$$\frac{d\boldsymbol{y}_{504}}{dt} = \left(1 \cdot \boldsymbol{k}_{on,comp}\boldsymbol{C}_C\boldsymbol{y}_{513} - 4 \cdot \boldsymbol{k}_{off,comp}\boldsymbol{y}_{504}\right) + \left(4 \cdot \frac{1}{5} \cdot \boldsymbol{k}_{on}\boldsymbol{c}_{eff}\boldsymbol{y}_{514} - 2 \cdot \boldsymbol{k}_{off}\boldsymbol{y}_{504}\right)$$

3rd row (three gC1q heads bound):

$$\frac{d\boldsymbol{y}_{530}}{dt} = -\left(3 \cdot \boldsymbol{k}_{on,comp}\boldsymbol{C}_C\boldsymbol{y}_{530} - 1 \cdot \boldsymbol{k}_{off,comp}\boldsymbol{y}_{521}\right) - \left(2 \cdot \frac{3}{3} \cdot \boldsymbol{k}_{on}\boldsymbol{c}_{eff}\boldsymbol{y}_{530} - 4 \cdot \boldsymbol{k}_{off}\boldsymbol{y}_{520}\right) + \left(3 \cdot \frac{4}{4} \cdot \boldsymbol{k}_{on}\boldsymbol{c}_{eff}\boldsymbol{y}_{540} - 3 \cdot \boldsymbol{k}_{off}\boldsymbol{y}_{530}\right)$$

$$\frac{d\boldsymbol{y}_{521}}{dt} = \left(3 \cdot \boldsymbol{k}_{on,comp}\boldsymbol{C}_C\boldsymbol{y}_{530} - 1 \cdot \boldsymbol{k}_{off,comp}\boldsymbol{y}_{521}\right) - \left(2 \cdot \boldsymbol{k}_{on,comp}\boldsymbol{C}_C\boldsymbol{y}_{521} - 2 \cdot \boldsymbol{k}_{off,comp}\boldsymbol{y}_{512}\right) - \left(2 \cdot \frac{2}{3} \cdot \boldsymbol{k}_{on}\boldsymbol{c}_{eff}\boldsymbol{y}_{521} - 4 \cdot \boldsymbol{k}_{off}\boldsymbol{y}_{511}\right) + \left(3 \cdot \frac{3}{4} \cdot \boldsymbol{k}_{on}\boldsymbol{c}_{eff}\boldsymbol{y}_{531} - 3 \cdot \boldsymbol{k}_{off}\boldsymbol{y}_{521}\right)$$

$$\frac{d\boldsymbol{y}_{512}}{dt} = \left(2 \cdot \boldsymbol{k}_{on,comp}\boldsymbol{C}_C\boldsymbol{y}_{521} - 2 \cdot \boldsymbol{k}_{off,comp}\boldsymbol{y}_{512}\right) - \left(1 \cdot \boldsymbol{k}_{on,comp}\boldsymbol{C}_C\boldsymbol{y}_{512} - 3 \cdot \boldsymbol{k}_{off,comp}\boldsymbol{y}_{503}\right) - \left(2 \cdot \frac{1}{3} \cdot \boldsymbol{k}_{on}\boldsymbol{c}_{eff}\boldsymbol{y}_{512} - 4 \cdot \boldsymbol{k}_{off}\boldsymbol{y}_{502}\right) + \left(3 \cdot \frac{2}{4} \cdot \boldsymbol{k}_{on}\boldsymbol{c}_{eff}\boldsymbol{y}_{522} - 3 \cdot \boldsymbol{k}_{off}\boldsymbol{y}_{512}\right)$$

$$\frac{d\boldsymbol{y}_{503}}{dt} = \left(1 \cdot \boldsymbol{k}_{on,comp}\boldsymbol{C}_C\boldsymbol{y}_{512} - 3 \cdot \boldsymbol{k}_{off,comp}\boldsymbol{y}_{503}\right) + \left(3 \cdot \frac{1}{4} \cdot \boldsymbol{k}_{on}\boldsymbol{c}_{eff}\boldsymbol{y}_{513} - 3 \cdot \boldsymbol{k}_{off}\boldsymbol{y}_{503}\right)$$

4th row (four gC1q heads bound):

$$\frac{d\boldsymbol{y}_{520}}{dt} = -\left(2 \cdot \boldsymbol{k}_{on,comp}\boldsymbol{C}_C\boldsymbol{y}_{520} - 1 \cdot \boldsymbol{k}_{off,comp}\boldsymbol{y}_{511}\right) - \left(1 \cdot \frac{2}{2} \cdot \boldsymbol{k}_{on}\boldsymbol{c}_{eff}\boldsymbol{y}_{520} - 5 \cdot \boldsymbol{k}_{off}\boldsymbol{y}_{510}\right) + \left(2 \cdot \frac{3}{3} \cdot \boldsymbol{k}_{on}\boldsymbol{c}_{eff}\boldsymbol{y}_{530} - 4 \cdot \boldsymbol{k}_{off}\boldsymbol{y}_{520}\right)$$

$$\frac{d\boldsymbol{y}_{511}}{dt} = \left(2 \cdot \boldsymbol{k}_{on,comp}\boldsymbol{C}_C\boldsymbol{y}_{520} - 1 \cdot \boldsymbol{k}_{off,comp}\boldsymbol{y}_{511}\right) - \left(1 \cdot \boldsymbol{k}_{on,comp}\boldsymbol{C}_C\boldsymbol{y}_{511} - 2 \cdot \boldsymbol{k}_{off,comp}\boldsymbol{y}_{502}\right) - \left(1 \cdot \frac{1}{2} \cdot \boldsymbol{k}_{on}\boldsymbol{c}_{eff}\boldsymbol{y}_{511} - 5 \cdot \boldsymbol{k}_{off}\boldsymbol{y}_{501}\right) + \left(2 \cdot \frac{2}{3} \cdot \boldsymbol{k}_{on}\boldsymbol{c}_{eff}\boldsymbol{y}_{521} - 4 \cdot \boldsymbol{k}_{off}\boldsymbol{y}_{511}\right)$$

$$\frac{d\boldsymbol{y}_{502}}{dt} = \left(1 \cdot \boldsymbol{k}_{on,comp}\boldsymbol{C}_C\boldsymbol{y}_{511} - 2 \cdot \boldsymbol{k}_{off,comp}\boldsymbol{y}_{502}\right) + \left(2 \cdot \frac{1}{3} \cdot \boldsymbol{k}_{on}\boldsymbol{c}_{eff}\boldsymbol{y}_{512} - 4 \cdot \boldsymbol{k}_{off}\boldsymbol{y}_{502}\right)$$

5th row (five gC1q heads bound):

$$\frac{d\boldsymbol{y}_{510}}{dt} = -\left(1 \cdot \boldsymbol{k}_{on,comp}\boldsymbol{C}_C\boldsymbol{y}_{510} - 1 \cdot \boldsymbol{k}_{off,comp}\boldsymbol{y}_{501}\right) + \left(1 \cdot \frac{2}{2} \cdot \boldsymbol{k}_{on}\boldsymbol{c}_{eff}\boldsymbol{y}_{520} - 5 \cdot \boldsymbol{k}_{off}\boldsymbol{y}_{510}\right)$$

$$\frac{d\boldsymbol{y}_{501}}{dt} = \left(1 \cdot \boldsymbol{k}_{on,comp}\boldsymbol{C}_C\boldsymbol{y}_{510} - 1 \cdot \boldsymbol{k}_{off,comp}\boldsymbol{y}_{501}\right) + \left(1 \cdot \frac{1}{2} \cdot \boldsymbol{k}_{on}\boldsymbol{c}_{eff}\boldsymbol{y}_{511} - 5 \cdot \boldsymbol{k}_{off}\boldsymbol{y}_{501}\right)$$

Pentamers without C1/C1q:

$$\frac{d\boldsymbol{y}_5}{dt} = -30 \cdot \boldsymbol{k}_{on}\boldsymbol{C}_{C1}\boldsymbol{y}_5 + \boldsymbol{k}_{off}(\boldsymbol{y}_{550} + \boldsymbol{y}_{541} + \boldsymbol{y}_{532} + \boldsymbol{y}_{523} + \boldsymbol{y}_{514} + \boldsymbol{y}_{505})$$

III. C1/C1q binding to IgG Tetramers (Fig. S5C):

1st row (one gC1q head bound):

$$\frac{dy_{450}}{dt} = -\left(5\cdot k_{on,comp}C_C y_{450} - 1\cdot k_{off,comp}y_{441}\right) - \left(3\cdot\frac{5}{5}\cdot k_{on}c_{eff}y_{450} - 2\cdot k_{off}y_{440}\right) + \left(24\cdot k_{on}C_{C1}y_4 - k_{off}y_{450}\right)$$

$$\frac{dy_{441}}{dt} = \left(5\cdot k_{on,comp}C_C y_{450} - 1\cdot k_{off,comp}y_{441}\right) - \left(4\cdot k_{on,comp}C_C y_{441} - 2\cdot k_{off,comp}y_{432}\right) - \left(3\cdot\frac{4}{5}\cdot k_{on}c_{eff}y_{441} - 2\cdot k_{off}y_{431}\right) + \left(-k_{off}y_{441}\right)$$

$$\frac{dy_{432}}{dt} = \left(4\cdot k_{on,comp}C_C y_{441} - 2\cdot k_{off,comp}y_{432}\right) - \left(3\cdot k_{on,comp}C_C y_{432} - 3\cdot k_{off,comp}y_{423}\right) - \left(3\cdot\frac{3}{5}\cdot k_{on}c_{eff}y_{432} - 2\cdot k_{off}y_{422}\right) + \left(-k_{off}y_{432}\right)$$

$$\frac{dy_{423}}{dt} = \left(3\cdot k_{on,comp}C_C y_{432} - 3\cdot k_{off,comp}y_{423}\right) - \left(2\cdot k_{on,comp}C_C y_{423} - 4\cdot k_{off,comp}y_{414}\right) - \left(3\cdot\frac{2}{5}\cdot k_{on}c_{eff}y_{423} - 2\cdot k_{off}y_{413}\right) + \left(-k_{off}y_{423}\right)$$

$$\frac{dy_{414}}{dt} = \left(2\cdot k_{on,comp}C_C y_{423} - 4\cdot k_{off,comp}y_{414}\right) - \left(1\cdot k_{on,comp}C_C y_{414} - 5\cdot k_{off,comp}y_{405}\right) - \left(3\cdot\frac{1}{5}\cdot k_{on}c_{eff}y_{414} - 2\cdot k_{off}y_{404}\right) + \left(-k_{off}y_{414}\right)$$

$$\frac{dy_{405}}{dt} = \left(1\cdot k_{on,comp}C_C y_{414} - 5\cdot k_{off,comp}y_{405}\right) + \left(-k_{off}y_{405}\right)$$

2nd row (two gC1q heads bound):

$$\frac{dy_{440}}{dt} = -\left(4\cdot k_{on,comp}C_C y_{440} - 1\cdot k_{off,comp}y_{431}\right) - \left(2\cdot\frac{4}{4}\cdot k_{on}c_{eff}y_{440} - 3\cdot k_{off}y_{430}\right) + \left(3\cdot\frac{5}{5}\cdot k_{on}c_{eff}y_{450} - 2\cdot k_{off}y_{440}\right)$$

$$\frac{dy_{431}}{dt} = \left(4\cdot k_{on,comp}C_C y_{440} - 1\cdot k_{off,comp}y_{431}\right) - \left(3\cdot k_{on,comp}C_C y_{431} - 2\cdot k_{off,comp}y_{422}\right) - \left(2\cdot\frac{3}{4}\cdot k_{on}c_{eff}y_{431} - 3\cdot k_{off}y_{421}\right) + \left(3\cdot\frac{4}{5}\cdot k_{on}c_{eff}y_{441} - 2\cdot k_{off}y_{431}\right)$$

$$\frac{dy_{422}}{dt} = \left(3\cdot k_{on,comp}C_C y_{431} - 2\cdot k_{off,comp}y_{422}\right) - \left(2\cdot k_{on,comp}C_C y_{422} - 3\cdot k_{off,comp}y_{413}\right) - \left(2\cdot\frac{2}{4}\cdot k_{on}c_{eff}y_{422} - 3\cdot k_{off}y_{412}\right) + \left(3\cdot\frac{3}{5}\cdot k_{on}c_{eff}y_{432} - 2\cdot k_{off}y_{422}\right)$$

$$\frac{dy_{413}}{dt} = \left(2\cdot k_{on,comp}C_C y_{422} - 3\cdot k_{off,comp}y_{413}\right) - \left(1\cdot k_{on,comp}C_C y_{413} - 4\cdot k_{off,comp}y_{404}\right) - \left(2\cdot\frac{1}{4}\cdot k_{on}c_{eff}y_{413} - 3\cdot k_{off}y_{403}\right) + \left(3\cdot\frac{2}{5}\cdot k_{on}c_{eff}y_{423} - 2\cdot k_{off}y_{413}\right)$$

$$\frac{dy_{404}}{dt} = \left(1\cdot k_{on,comp}C_C y_{413} - 4\cdot k_{off,comp}y_{404}\right) + \left(3\cdot\frac{1}{5}\cdot k_{on}c_{eff}y_{414} - 2\cdot k_{off}y_{404}\right)$$

3rd row (three gC1q heads bound):

$$\frac{dy_{430}}{dt} = -\left(3\cdot k_{on,comp}C_C y_{430} - 1\cdot k_{off,comp}y_{421}\right) - \left(1\cdot\frac{3}{3}\cdot k_{on}c_{eff}y_{430} - 4\cdot k_{off}y_{420}\right) + \left(2\cdot\frac{4}{4}\cdot k_{on}c_{eff}y_{440} - 3\cdot k_{off}y_{430}\right)$$

$$\frac{dy_{421}}{dt} = \left(3\cdot k_{on,comp}C_C y_{430} - 1\cdot k_{off,comp}y_{421}\right) - \left(2\cdot k_{on,comp}C_C y_{421} - 2\cdot k_{off,comp}y_{412}\right) - \left(1\cdot\frac{2}{3}\cdot k_{on}c_{eff}y_{421} - 4\cdot k_{off}y_{411}\right) + \left(2\cdot\frac{3}{4}\cdot k_{on}c_{eff}y_{431} - 3\cdot k_{off}y_{421}\right)$$

$$\frac{dy_{412}}{dt} = \left(2\cdot k_{on,comp}C_C y_{421} - 2\cdot k_{off,comp}y_{412}\right) - \left(1\cdot k_{on,comp}C_C y_{412} - 3\cdot k_{off,comp}y_{403}\right) - \left(1\cdot\frac{1}{3}\cdot k_{on}c_{eff}y_{412} - 4\cdot k_{off}y_{402}\right) + \left(2\cdot\frac{2}{4}\cdot k_{on}c_{eff}y_{422} - 3\cdot k_{off}y_{412}\right)$$

$$\frac{dy_{403}}{dt} = \left(1\cdot k_{on,comp}C_C y_{412} - 3\cdot k_{off,comp}y_{403}\right) + \left(2\cdot\frac{1}{4}\cdot k_{on}c_{eff}y_{413} - 3\cdot k_{off}y_{403}\right)$$

4th row (four gC1q heads bound):

$$\frac{dy_{420}}{dt} = -\left(2\cdot k_{on,comp}C_C y_{420} - 1\cdot k_{off,comp}y_{411}\right) + \left(1\cdot\frac{3}{3}\cdot k_{on}c_{eff}y_{430} - 4\cdot k_{off}y_{420}\right)$$

$$\frac{d\boldsymbol{y}_{411}}{dt} = \left(2\cdot \boldsymbol{k}_{on,comp}\boldsymbol{C}_C\boldsymbol{y}_{420} - 1\cdot \boldsymbol{k}_{off,comp}\boldsymbol{y}_{411}\right) - \left(1\cdot \boldsymbol{k}_{on,comp}\boldsymbol{C}_C\boldsymbol{y}_{411} - 2\cdot \boldsymbol{k}_{off,comp}\boldsymbol{y}_{402}\right) + \left(1\cdot\frac{2}{3}\cdot \boldsymbol{k}_{on}\boldsymbol{c}_{eff}\boldsymbol{y}_{421} - 4\cdot \boldsymbol{k}_{off}\boldsymbol{y}_{411}\right)$$

$$\frac{d\boldsymbol{y}_{402}}{dt} = \left(1\cdot \boldsymbol{k}_{on,comp}\boldsymbol{C}_C\boldsymbol{y}_{411} - 2\cdot \boldsymbol{k}_{off,comp}\boldsymbol{y}_{402}\right) + \left(1\cdot\frac{1}{3}\cdot \boldsymbol{k}_{on}\boldsymbol{c}_{eff}\boldsymbol{y}_{412} - 4\cdot \boldsymbol{k}_{off}\boldsymbol{y}_{402}\right)$$

Tetramers without C1/C1q:

$$\frac{d\boldsymbol{y}_{4}}{dt} = -24\cdot \boldsymbol{k}_{on}\boldsymbol{C}_{C1}\boldsymbol{y}_4 + \boldsymbol{k}_{off}\left(\boldsymbol{y}_{450} + \boldsymbol{y}_{441} + \boldsymbol{y}_{432} + \boldsymbol{y}_{423} + \boldsymbol{y}_{414} + \boldsymbol{y}_{405}\right)$$

IV. C1/C1q binding to IgG Trimers (Fig. S5D):

1st row (one gC1q head bound):

$$\frac{d\boldsymbol{y}_{350}}{dt} = -\left(5\cdot \boldsymbol{k}_{on,comp}\boldsymbol{C}_C\boldsymbol{y}_{350} - 1\cdot \boldsymbol{k}_{off,comp}\boldsymbol{y}_{341}\right) - \left(2\cdot\frac{5}{5}\cdot \boldsymbol{k}_{on}\boldsymbol{c}_{eff}\boldsymbol{y}_{350} - 2\cdot \boldsymbol{k}_{off}\boldsymbol{y}_{340}\right) + \left(18\cdot \boldsymbol{k}_{on}\boldsymbol{C}_{C1}\boldsymbol{y}_3 - \boldsymbol{k}_{off}\boldsymbol{y}_{350}\right)$$

$$\frac{d\boldsymbol{y}_{341}}{dt} = \left(5\cdot \boldsymbol{k}_{on,comp}\boldsymbol{C}_C\boldsymbol{y}_{350} - 1\cdot \boldsymbol{k}_{off,comp}\boldsymbol{y}_{341}\right) - \left(4\cdot \boldsymbol{k}_{on,comp}\boldsymbol{C}_C\boldsymbol{y}_{341} - 2\cdot \boldsymbol{k}_{off,comp}\boldsymbol{y}_{332}\right) - \left(2\cdot\frac{4}{5}\cdot \boldsymbol{k}_{on}\boldsymbol{c}_{eff}\boldsymbol{y}_{341} - 2\cdot \boldsymbol{k}_{off}\boldsymbol{y}_{331}\right) + \left(-\boldsymbol{k}_{off}\boldsymbol{y}_{341}\right)$$

$$\frac{d\boldsymbol{y}_{332}}{dt} = \left(4\cdot \boldsymbol{k}_{on,comp}\boldsymbol{C}_C\boldsymbol{y}_{341} - 2\cdot \boldsymbol{k}_{off,comp}\boldsymbol{y}_{332}\right) - \left(3\cdot \boldsymbol{k}_{on,comp}\boldsymbol{C}_C\boldsymbol{y}_{332} - 3\cdot \boldsymbol{k}_{off,comp}\boldsymbol{y}_{323}\right) - \left(2\cdot\frac{3}{5}\cdot \boldsymbol{k}_{on}\boldsymbol{c}_{eff}\boldsymbol{y}_{332} - 2\cdot \boldsymbol{k}_{off}\boldsymbol{y}_{322}\right) + \left(-\boldsymbol{k}_{off}\boldsymbol{y}_{332}\right)$$

$$\frac{d\boldsymbol{y}_{323}}{dt} = \left(3\cdot \boldsymbol{k}_{on,comp}\boldsymbol{C}_C\boldsymbol{y}_{332} - 3\cdot \boldsymbol{k}_{off,comp}\boldsymbol{y}_{323}\right) - \left(2\cdot \boldsymbol{k}_{on,comp}\boldsymbol{C}_C\boldsymbol{y}_{323} - 4\cdot \boldsymbol{k}_{off,comp}\boldsymbol{y}_{314}\right) - \left(2\cdot\frac{2}{5}\cdot \boldsymbol{k}_{on}\boldsymbol{c}_{eff}\boldsymbol{y}_{323} - 2\cdot \boldsymbol{k}_{off}\boldsymbol{y}_{313}\right) + \left(-\boldsymbol{k}_{off}\boldsymbol{y}_{323}\right)$$

$$\frac{d\boldsymbol{y}_{314}}{dt} = \left(2\cdot \boldsymbol{k}_{on,comp}\boldsymbol{C}_C\boldsymbol{y}_{323} - 4\cdot \boldsymbol{k}_{off,comp}\boldsymbol{y}_{314}\right) - \left(1\cdot \boldsymbol{k}_{on,comp}\boldsymbol{C}_C\boldsymbol{y}_{314} - 5\cdot \boldsymbol{k}_{off,comp}\boldsymbol{y}_{305}\right) - \left(2\cdot\frac{1}{5}\cdot \boldsymbol{k}_{on}\boldsymbol{c}_{eff}\boldsymbol{y}_{314} - 2\cdot \boldsymbol{k}_{off}\boldsymbol{y}_{304}\right) + \left(-\boldsymbol{k}_{off}\boldsymbol{y}_{314}\right)$$

$$\frac{d\boldsymbol{y}_{305}}{dt} = \left(1\cdot \boldsymbol{k}_{on,comp}\boldsymbol{C}_C\boldsymbol{y}_{314} - 5\cdot \boldsymbol{k}_{off,comp}\boldsymbol{y}_{305}\right) + \left(-\boldsymbol{k}_{off}\boldsymbol{y}_{305}\right)$$

2nd row (two gC1q heads bound):

$$\frac{d\boldsymbol{y}_{340}}{dt} = -\left(4\cdot \boldsymbol{k}_{on,comp}\boldsymbol{C}_C\boldsymbol{y}_{340} - 1\cdot \boldsymbol{k}_{off,comp}\boldsymbol{y}_{331}\right) - \left(1\cdot\frac{4}{4}\cdot \boldsymbol{k}_{on}\boldsymbol{c}_{eff}\boldsymbol{y}_{340} - 3\cdot \boldsymbol{k}_{off}\boldsymbol{y}_{330}\right) + \left(2\cdot\frac{5}{5}\cdot \boldsymbol{k}_{on}\boldsymbol{c}_{eff}\boldsymbol{y}_{350} - 2\cdot \boldsymbol{k}_{off}\boldsymbol{y}_{340}\right)$$

$$\frac{d\boldsymbol{y}_{331}}{dt} = \left(4\cdot \boldsymbol{k}_{on,comp}\boldsymbol{C}_C\boldsymbol{y}_{340} - 1\cdot \boldsymbol{k}_{off,comp}\boldsymbol{y}_{331}\right) - \left(3\cdot \boldsymbol{k}_{on,comp}\boldsymbol{C}_C\boldsymbol{y}_{331} - 2\cdot \boldsymbol{k}_{off,comp}\boldsymbol{y}_{322}\right) - \left(1\cdot\frac{3}{4}\cdot \boldsymbol{k}_{on}\boldsymbol{c}_{eff}\boldsymbol{y}_{331} - 3\cdot \boldsymbol{k}_{off}\boldsymbol{y}_{321}\right) + \left(2\cdot\frac{4}{5}\cdot \boldsymbol{k}_{on}\boldsymbol{c}_{eff}\boldsymbol{y}_{341} - 2\cdot \boldsymbol{k}_{off}\boldsymbol{y}_{331}\right)$$

$$\frac{d\boldsymbol{y}_{322}}{dt} = \left(3\cdot \boldsymbol{k}_{on,comp}\boldsymbol{C}_C\boldsymbol{y}_{331} - 2\cdot \boldsymbol{k}_{off,comp}\boldsymbol{y}_{322}\right) - \left(2\cdot \boldsymbol{k}_{on,comp}\boldsymbol{C}_C\boldsymbol{y}_{322} - 3\cdot \boldsymbol{k}_{off,comp}\boldsymbol{y}_{313}\right) - \left(1\cdot\frac{2}{4}\cdot \boldsymbol{k}_{on}\boldsymbol{c}_{eff}\boldsymbol{y}_{322} - 3\cdot \boldsymbol{k}_{off}\boldsymbol{y}_{312}\right) + \left(2\cdot\frac{3}{5}\cdot \boldsymbol{k}_{on}\boldsymbol{c}_{eff}\boldsymbol{y}_{332} - 2\cdot \boldsymbol{k}_{off}\boldsymbol{y}_{322}\right)$$

$$\frac{d\boldsymbol{y}_{313}}{dt} = \left(2\cdot \boldsymbol{k}_{on,comp}\boldsymbol{C}_C\boldsymbol{y}_{322} - 3\cdot \boldsymbol{k}_{off,comp}\boldsymbol{y}_{313}\right) - \left(1\cdot \boldsymbol{k}_{on,comp}\boldsymbol{C}_C\boldsymbol{y}_{313} - 4\cdot \boldsymbol{k}_{off,comp}\boldsymbol{y}_{304}\right) - \left(1\cdot\frac{1}{4}\cdot \boldsymbol{k}_{on}\boldsymbol{c}_{eff}\boldsymbol{y}_{313} - 3\cdot \boldsymbol{k}_{off}\boldsymbol{y}_{303}\right) + \left(2\cdot\frac{2}{5}\cdot \boldsymbol{k}_{on}\boldsymbol{c}_{eff}\boldsymbol{y}_{323} - 2\cdot \boldsymbol{k}_{off}\boldsymbol{y}_{313}\right)$$

$$\frac{d\boldsymbol{y}_{304}}{dt} = \left(1\cdot \boldsymbol{k}_{on,comp}\boldsymbol{C}_C\boldsymbol{y}_{313} - 4\cdot \boldsymbol{k}_{off,comp}\boldsymbol{y}_{304}\right) + \left(2\cdot\frac{1}{5}\cdot \boldsymbol{k}_{on}\boldsymbol{c}_{eff}\boldsymbol{y}_{314} - 2\cdot \boldsymbol{k}_{off}\boldsymbol{y}_{304}\right)$$

3rd row (three gC1q heads bound):

$$\frac{d\boldsymbol{y}_{330}}{dt} = -\left(3\cdot \boldsymbol{k}_{on,comp}\boldsymbol{C}_C\boldsymbol{y}_{330} - 1\cdot \boldsymbol{k}_{off,comp}\boldsymbol{y}_{321}\right) + \left(1\cdot\frac{4}{4}\cdot \boldsymbol{k}_{on}\boldsymbol{c}_{eff}\boldsymbol{y}_{340} - 3\cdot \boldsymbol{k}_{off}\boldsymbol{y}_{330}\right)$$
$$\frac{d\boldsymbol{y}_{321}}{dt} = \left(3\cdot \boldsymbol{k}_{on,comp}\boldsymbol{C}_C\boldsymbol{y}_{330} - 1\cdot \boldsymbol{k}_{off,comp}\boldsymbol{y}_{321}\right) - \left(2\cdot \boldsymbol{k}_{on,comp}\boldsymbol{C}_C\boldsymbol{y}_{321} - 2\cdot \boldsymbol{k}_{off,comp}\boldsymbol{y}_{312}\right) + \left(1\cdot\frac{3}{4}\cdot \boldsymbol{k}_{on}\boldsymbol{c}_{eff}\boldsymbol{y}_{331} - 3\cdot \boldsymbol{k}_{off}\boldsymbol{y}_{321}\right)$$
$$\frac{d\boldsymbol{y}_{312}}{dt} = \left(2\cdot \boldsymbol{k}_{on,comp}\boldsymbol{C}_C\boldsymbol{y}_{321} - 2\cdot \boldsymbol{k}_{off,comp}\boldsymbol{y}_{312}\right) - \left(1\cdot \boldsymbol{k}_{on,comp}\boldsymbol{C}_C\boldsymbol{y}_{312} - 3\cdot \boldsymbol{k}_{off,comp}\boldsymbol{y}_{303}\right) + \left(1\cdot\frac{2}{4}\cdot \boldsymbol{k}_{on}\boldsymbol{c}_{eff}\boldsymbol{y}_{322} - 3\cdot \boldsymbol{k}_{off}\boldsymbol{y}_{312}\right)$$
$$\frac{d\boldsymbol{y}_{303}}{dt} = \left(1\cdot \boldsymbol{k}_{on,comp}\boldsymbol{C}_C\boldsymbol{y}_{312} - 3\cdot \boldsymbol{k}_{off,comp}\boldsymbol{y}_{303}\right) + \left(1\cdot\frac{1}{4}\cdot \boldsymbol{k}_{on}\boldsymbol{c}_{eff}\boldsymbol{y}_{313} - 3\cdot \boldsymbol{k}_{off}\boldsymbol{y}_{303}\right)$$

Trimers without C1/C1q:

$$\frac{d\boldsymbol{y}_{3}}{dt} = -18\cdot \boldsymbol{k}_{on}\boldsymbol{C}_{C1}\boldsymbol{y}_3 + \boldsymbol{k}_{off}\left(\boldsymbol{y}_{350} + \boldsymbol{y}_{341} + \boldsymbol{y}_{332} + \boldsymbol{y}_{323} + \boldsymbol{y}_{314} + \boldsymbol{y}_{305}\right)$$

V. C1/C1q binding to IgG Dimers (Fig. S5E):

1st row (one gC1q head bound):

$$\frac{d\boldsymbol{y}_{250}}{dt} = -\left(5\cdot \boldsymbol{k}_{on,comp}\boldsymbol{C}_C\boldsymbol{y}_{250} - 1\cdot \boldsymbol{k}_{off,comp}\boldsymbol{y}_{241}\right) - \left(1\cdot\frac{5}{5}\cdot \boldsymbol{k}_{on}\boldsymbol{c}_{eff}\boldsymbol{y}_{250} - 2\cdot \boldsymbol{k}_{off}\boldsymbol{y}_{240}\right) + \left(12\cdot \boldsymbol{k}_{on}\boldsymbol{C}_{C1}\boldsymbol{y}_2 - \boldsymbol{k}_{off}\boldsymbol{y}_{250}\right)$$
$$\frac{d\boldsymbol{y}_{241}}{dt} = \left(5\cdot \boldsymbol{k}_{on,comp}\boldsymbol{C}_C\boldsymbol{y}_{250} - 1\cdot \boldsymbol{k}_{off,comp}\boldsymbol{y}_{241}\right) - \left(4\cdot \boldsymbol{k}_{on,comp}\boldsymbol{C}_C\boldsymbol{y}_{241} - 2\cdot \boldsymbol{k}_{off,comp}\boldsymbol{y}_{232}\right) - \left(1\cdot\frac{4}{5}\cdot \boldsymbol{k}_{on}\boldsymbol{c}_{eff}\boldsymbol{y}_{241} - 2\cdot \boldsymbol{k}_{off}\boldsymbol{y}_{231}\right) + \left(-\boldsymbol{k}_{off}\boldsymbol{y}_{241}\right)$$
$$\frac{d\boldsymbol{y}_{232}}{dt} = \left(4\cdot \boldsymbol{k}_{on,comp}\boldsymbol{C}_C\boldsymbol{y}_{241} - 2\cdot \boldsymbol{k}_{off,comp}\boldsymbol{y}_{232}\right) - \left(3\cdot \boldsymbol{k}_{on,comp}\boldsymbol{C}_C\boldsymbol{y}_{232} - 3\cdot \boldsymbol{k}_{off,comp}\boldsymbol{y}_{223}\right) - \left(1\cdot\frac{3}{5}\cdot \boldsymbol{k}_{on}\boldsymbol{c}_{eff}\boldsymbol{y}_{232} - 2\cdot \boldsymbol{k}_{off}\boldsymbol{y}_{222}\right) + \left(-\boldsymbol{k}_{off}\boldsymbol{y}_{232}\right)$$
$$\frac{d\boldsymbol{y}_{223}}{dt} = \left(3\cdot \boldsymbol{k}_{on,comp}\boldsymbol{C}_C\boldsymbol{y}_{232} - 3\cdot \boldsymbol{k}_{off,comp}\boldsymbol{y}_{223}\right) - \left(2\cdot \boldsymbol{k}_{on,comp}\boldsymbol{C}_C\boldsymbol{y}_{223} - 4\cdot \boldsymbol{k}_{off,comp}\boldsymbol{y}_{214}\right) - \left(1\cdot\frac{2}{5}\cdot \boldsymbol{k}_{on}\boldsymbol{c}_{eff}\boldsymbol{y}_{223} - 2\cdot \boldsymbol{k}_{off}\boldsymbol{y}_{213}\right) + \left(-\boldsymbol{k}_{off}\boldsymbol{y}_{223}\right)$$
$$\frac{d\boldsymbol{y}_{214}}{dt} = \left(2\cdot \boldsymbol{k}_{on,comp}\boldsymbol{C}_C\boldsymbol{y}_{223} - 4\cdot \boldsymbol{k}_{off,comp}\boldsymbol{y}_{214}\right) - \left(1\cdot \boldsymbol{k}_{on,comp}\boldsymbol{C}_C\boldsymbol{y}_{214} - 5\cdot \boldsymbol{k}_{off,comp}\boldsymbol{y}_{205}\right) - \left(1\cdot\frac{1}{5}\cdot \boldsymbol{k}_{on}\boldsymbol{c}_{eff}\boldsymbol{y}_{214} - 2\cdot \boldsymbol{k}_{off}\boldsymbol{y}_{204}\right) + \left(-\boldsymbol{k}_{off}\boldsymbol{y}_{214}\right)$$
$$\frac{d\boldsymbol{y}_{205}}{dt} = \left(1\cdot \boldsymbol{k}_{on,comp}\boldsymbol{C}_C\boldsymbol{y}_{214} - 5\cdot \boldsymbol{k}_{off,comp}\boldsymbol{y}_{205}\right) + \left(-\boldsymbol{k}_{off}\boldsymbol{y}_{205}\right)$$

2nd row (two gC1q heads bound):

$$\frac{d\boldsymbol{y}_{240}}{dt} = -\left(4\cdot \boldsymbol{k}_{on,comp}\boldsymbol{C}_C\boldsymbol{y}_{240} - 1\cdot \boldsymbol{k}_{off,comp}\boldsymbol{y}_{31}\right) - + \left(1\cdot\frac{5}{5}\cdot \boldsymbol{k}_{on}\boldsymbol{c}_{eff}\boldsymbol{y}_{250} - 2\cdot \boldsymbol{k}_{off}\boldsymbol{y}_{240}\right)$$
$$\frac{d\boldsymbol{y}_{231}}{dt} = \left(4\cdot \boldsymbol{k}_{on,comp}\boldsymbol{C}_C\boldsymbol{y}_{240} - 1\cdot \boldsymbol{k}_{off,comp}\boldsymbol{y}_{231}\right) - \left(3\cdot \boldsymbol{k}_{on,comp}\boldsymbol{C}_C\boldsymbol{y}_{231} - 2\cdot \boldsymbol{k}_{off,comp}\boldsymbol{y}_{222}\right) - + \left(1\cdot\frac{4}{5}\cdot \boldsymbol{k}_{on}\boldsymbol{c}_{eff}\boldsymbol{y}_{241} - 2\cdot \boldsymbol{k}_{off}\boldsymbol{y}_{231}\right)$$
$$\frac{d\boldsymbol{y}_{222}}{dt} = \left(3\cdot \boldsymbol{k}_{on,comp}\boldsymbol{C}_C\boldsymbol{y}_{231} - 2\cdot \boldsymbol{k}_{off,comp}\boldsymbol{y}_{222}\right) - \left(2\cdot \boldsymbol{k}_{on,comp}\boldsymbol{C}_C\boldsymbol{y}_{222} - 3\cdot \boldsymbol{k}_{off,comp}\boldsymbol{y}_{213}\right) - + \left(1\cdot\frac{3}{5}\cdot \boldsymbol{k}_{on}\boldsymbol{c}_{eff}\boldsymbol{y}_{232} - 2\cdot \boldsymbol{k}_{off}\boldsymbol{y}_{222}\right)$$
$$\frac{d\boldsymbol{y}_{213}}{dt} = \left(2\cdot \boldsymbol{k}_{on,comp}\boldsymbol{C}_C\boldsymbol{y}_{222} - 3\cdot \boldsymbol{k}_{off,comp}\boldsymbol{y}_{213}\right) - \left(1\cdot \boldsymbol{k}_{on,comp}\boldsymbol{C}_C\boldsymbol{y}_{213} - 4\cdot \boldsymbol{k}_{off,comp}\boldsymbol{y}_{204}\right) - + \left(1\cdot\frac{2}{5}\cdot \boldsymbol{k}_{on}\boldsymbol{c}_{eff}\boldsymbol{y}_{223} - 2\cdot \boldsymbol{k}_{off}\boldsymbol{y}_{213}\right)$$

$$\frac{d\boldsymbol{y}_{204}}{dt} = \left(1 \cdot \boldsymbol{k}_{on,comp}\boldsymbol{C}_C\boldsymbol{y}_{213} - 4 \cdot \boldsymbol{k}_{off,comp}\boldsymbol{y}_{204}\right) \qquad + \left(1 \cdot \frac{1}{5} \cdot \boldsymbol{k}_{on}\boldsymbol{c}_{eff}\boldsymbol{y}_{214} - 2 \cdot \boldsymbol{k}_{off}\boldsymbol{y}_{204}\right)$$

Dimers without C1/C1q:

$$\frac{d\boldsymbol{y}_2}{dt} = -12 \cdot \boldsymbol{k}_{on}\boldsymbol{C}_{C1}\boldsymbol{y}_2 + \boldsymbol{k}_{off}(\boldsymbol{y}_{250} + \boldsymbol{y}_{241} + \boldsymbol{y}_{232} + \boldsymbol{y}_{223} + \boldsymbol{y}_{214} + \boldsymbol{y}_{205})$$

VI. C1/C1q binding to IgG Monomers (Fig. S5F):

1st row (one gC1q head bound):

$$\frac{d\boldsymbol{y}_{150}}{dt} = \qquad -\left(5 \cdot \boldsymbol{k}_{on,comp}\boldsymbol{C}_C\boldsymbol{y}_{150} - 1 \cdot \boldsymbol{k}_{off,comp}\boldsymbol{y}_{141}\right) - \qquad +(6 \cdot \boldsymbol{k}_{on}\boldsymbol{C}_{C1}\boldsymbol{y}_1 - \boldsymbol{k}_{off}\boldsymbol{y}_{150})$$

$$\frac{d\boldsymbol{y}_{141}}{dt} = \left(5 \cdot \boldsymbol{k}_{on,comp}\boldsymbol{C}_C\boldsymbol{y}_{150} - 1 \cdot \boldsymbol{k}_{off,comp}\boldsymbol{y}_{141}\right) - \left(4 \cdot \boldsymbol{k}_{on,comp}\boldsymbol{C}_C\boldsymbol{y}_{141} - 2 \cdot \boldsymbol{k}_{off,comp}\boldsymbol{y}_{132}\right) - \qquad +(\qquad -\boldsymbol{k}_{off}\boldsymbol{y}_{141})$$

$$\frac{d\boldsymbol{y}_{132}}{dt} = \left(4 \cdot \boldsymbol{k}_{on,comp}\boldsymbol{C}_C\boldsymbol{y}_{141} - 2 \cdot \boldsymbol{k}_{off,comp}\boldsymbol{y}_{132}\right) - \left(3 \cdot \boldsymbol{k}_{on,comp}\boldsymbol{C}_C\boldsymbol{y}_{132} - 3 \cdot \boldsymbol{k}_{off,comp}\boldsymbol{y}_{123}\right) - \qquad +(\qquad -\boldsymbol{k}_{off}\boldsymbol{y}_{132})$$

$$\frac{d\boldsymbol{y}_{123}}{dt} = \left(3 \cdot \boldsymbol{k}_{on,comp}\boldsymbol{C}_C\boldsymbol{y}_{132} - 3 \cdot \boldsymbol{k}_{off,comp}\boldsymbol{y}_{123}\right) - \left(2 \cdot \boldsymbol{k}_{on,comp}\boldsymbol{C}_C\boldsymbol{y}_{123} - 4 \cdot \boldsymbol{k}_{off,comp}\boldsymbol{y}_{114}\right) - \qquad +(\qquad -\boldsymbol{k}_{off}\boldsymbol{y}_{123})$$

$$\frac{d\boldsymbol{y}_{114}}{dt} = \left(2 \cdot \boldsymbol{k}_{on,comp}\boldsymbol{C}_C\boldsymbol{y}_{123} - 4 \cdot \boldsymbol{k}_{off,comp}\boldsymbol{y}_{114}\right) - \left(1 \cdot \boldsymbol{k}_{on,comp}\boldsymbol{C}_C\boldsymbol{y}_{114} - 5 \cdot \boldsymbol{k}_{off,comp}\boldsymbol{y}_{105}\right) - \qquad +(\qquad -\boldsymbol{k}_{off}\boldsymbol{y}_{114})$$

$$\frac{d\boldsymbol{y}_{105}}{dt} = \left(1 \cdot \boldsymbol{k}_{on,comp}\boldsymbol{C}_C\boldsymbol{y}_{114} - 5 \cdot \boldsymbol{k}_{off,comp}\boldsymbol{y}_{105}\right) \qquad + (\qquad -\boldsymbol{k}_{off}\boldsymbol{y}_{105})$$

Monomers without C1/C1q:

$$\frac{d\boldsymbol{y}_1}{dt} = -6 \cdot \boldsymbol{k}_{on}\boldsymbol{C}_{C1}\boldsymbol{y}_1 + \boldsymbol{k}_{off}(\boldsymbol{y}_{150} + \boldsymbol{y}_{141} + \boldsymbol{y}_{132} + \boldsymbol{y}_{123} + \boldsymbol{y}_{114} + \boldsymbol{y}_{105})$$

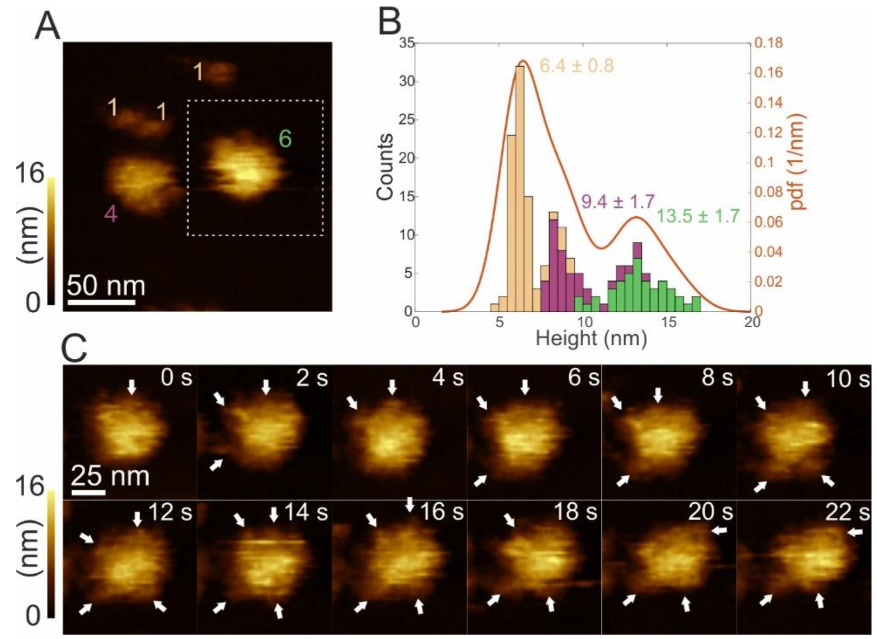

Figure S1. Structural comparison of IgG3 and IgG1 hexamers based on higher-resolution HS-AFM images. **(A)** First frame of HS-AFM movie S2 of one IgG3 hexamer, one tetramer and some monomers bound to a DNP-SLB. **(B)** Height histogram generated from height over time recordings of the oligomers in movie S2. Contributions of the respective oligomers are color coded according to (A). Numbers correspond to means ± s.d. over all image frames and individual particles, respectively. **(C)** High resolution images of an individual IgG3 hexamer (dashed area from (A)). Additional smaller structures surrounding the central Fc platform are indicated by arrows.

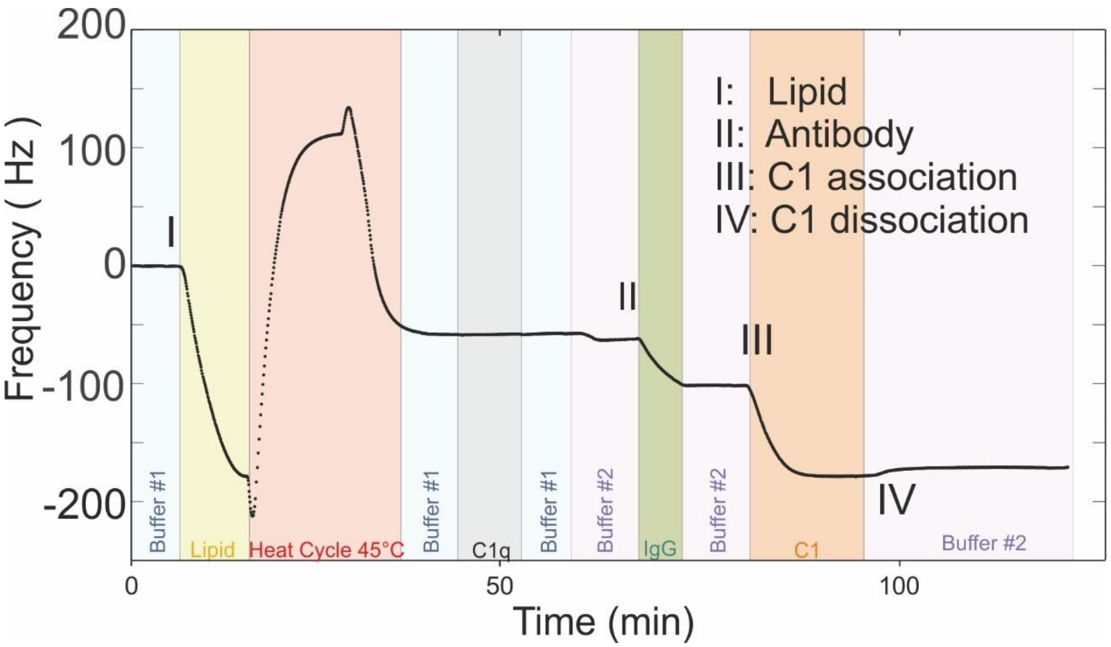

Figure S2. Typical QCM sensorgram of C1/C1q binding experiments. After equilibration with running buffer (buffer #1), a lipid suspension (200 µg/ml) of DNP-labeled liposomes is injected into the QCM liquid cell (I) until saturation is reached, followed by a heat cycle to a maximum temperature of 45° C to facilitate liposome spreading and fusion on the SiO_2-coated QCM crystal forming a dense DNP-SLB. After removing excess lipids by flushing the DNP-SLB with the running buffer #1, C1q is added at 15 nM to check for lipid membrane integrity (C1q would strongly associate with the bare SiO_2 surface when not covered by a lipid membrane). When no C1q binding was observed, the running buffer #1 was changed to buffer #2, and an anti-DNP IgG suspension (33 nM) was introduced (II) until the desired antibody density was reached. After removal of solution-phase IgGs through flushing with buffer #2, C1 or C1q was added at 15 nM until equilibrium was reached, after which a dissociation phase in buffer #2 was added (IV). Data shown corresponds to the third overtone f_3.

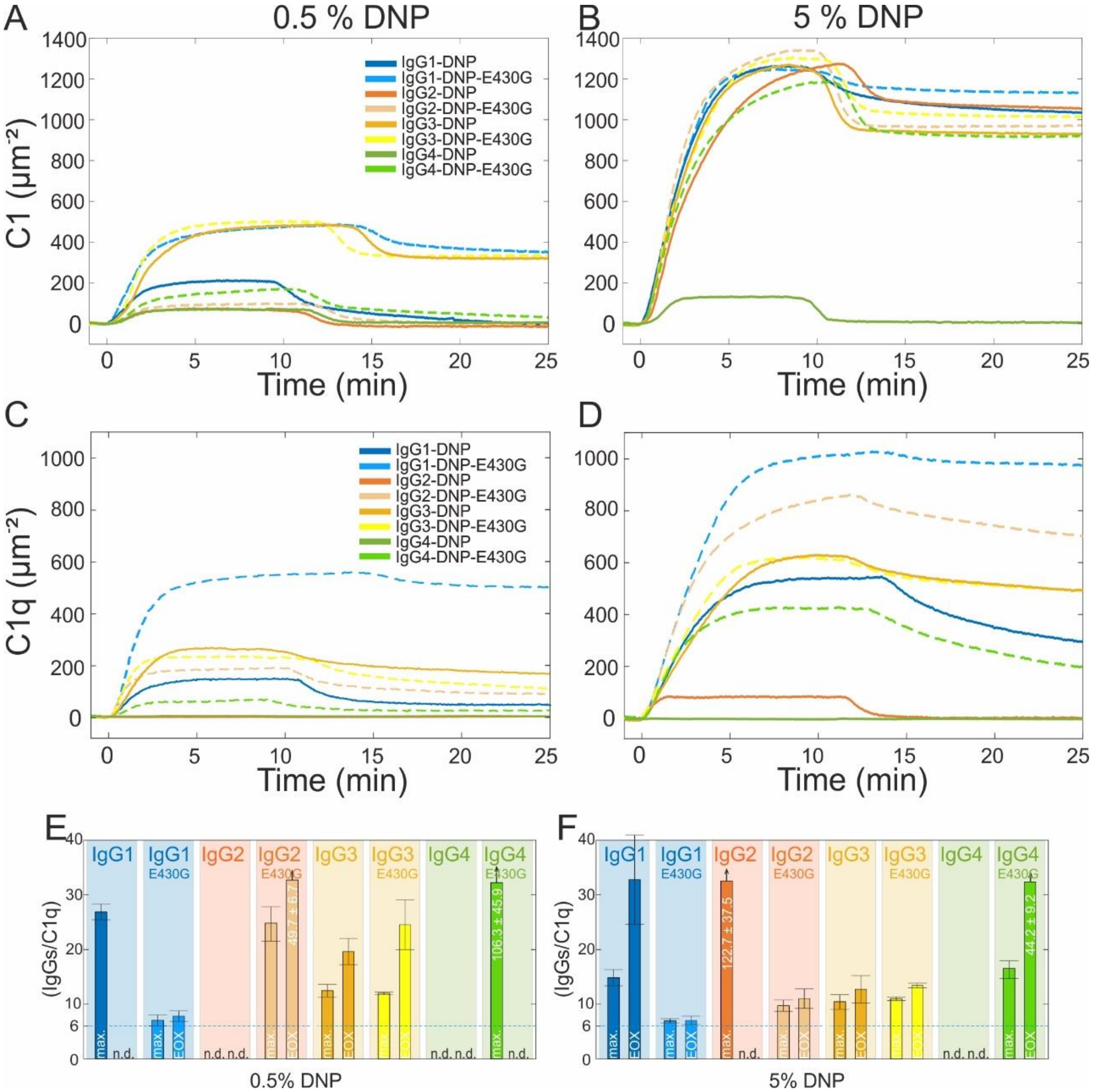

Figure S3. QCM sensorgrams of C1/C1q binding to IgG subclass variants and impact of antigen surface density on C1q recruitment efficiencies. **(A-B)** C1 binding to IgG subclass variants bound to 0.5 mol% DNP-SLBs (A) and 5 mol% DNP-SLBs (B). **(C-D)** C1q binding to IgG subclass variants bound to 0.5 mol% DNP-SLBs (C) and 5 mol% DNP-SLBs (D). **(E)** C1q recruitment efficiencies obtained for a medium antigen density of 0.5 mol% DNP-labeled lipids in the DNP-SLB. **(F)** C1q recruitment efficiencies of IgG1 and IgG3 variants obtained for a high antigen density of 5 mol% DNP-labeled lipids in the DNP-SLB. Depicted recruitment efficiencies are means ± s.d..

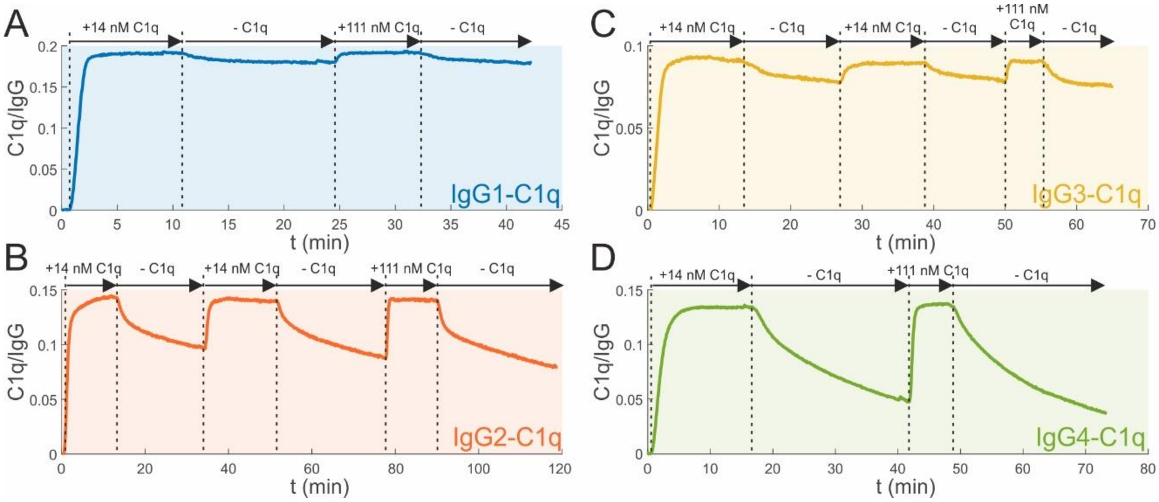

Figure S4. C1q binding to IgG-DNP-RGY opsonized DNP-SLBs saturates at the used C1q concentrations. **(A)** IgG1-DNP-RGY, **(B)** IgG2-DNP-RGY, **(C)** IgG3-DNP-RGY, **(D)** and IgG4-DNP-RGY. No significant additional binding was observed when 8-fold increasing the C1q concentration in the running buffer.

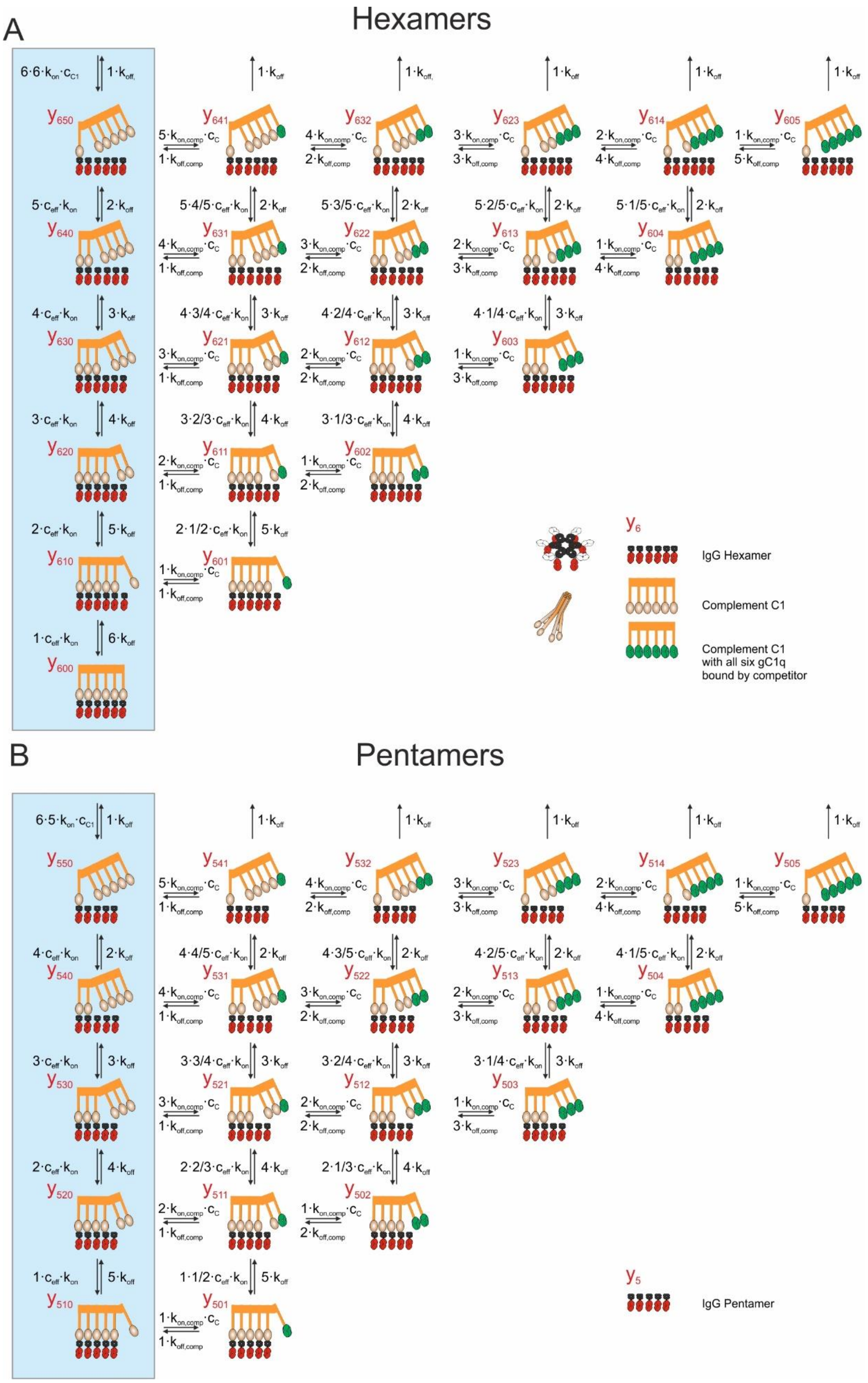
A
Hexamers
IgG Hexamer
Complement C1
Complement C1
with all six gC1q
bound by competitor
B
Pentamers
IgG Pentamer

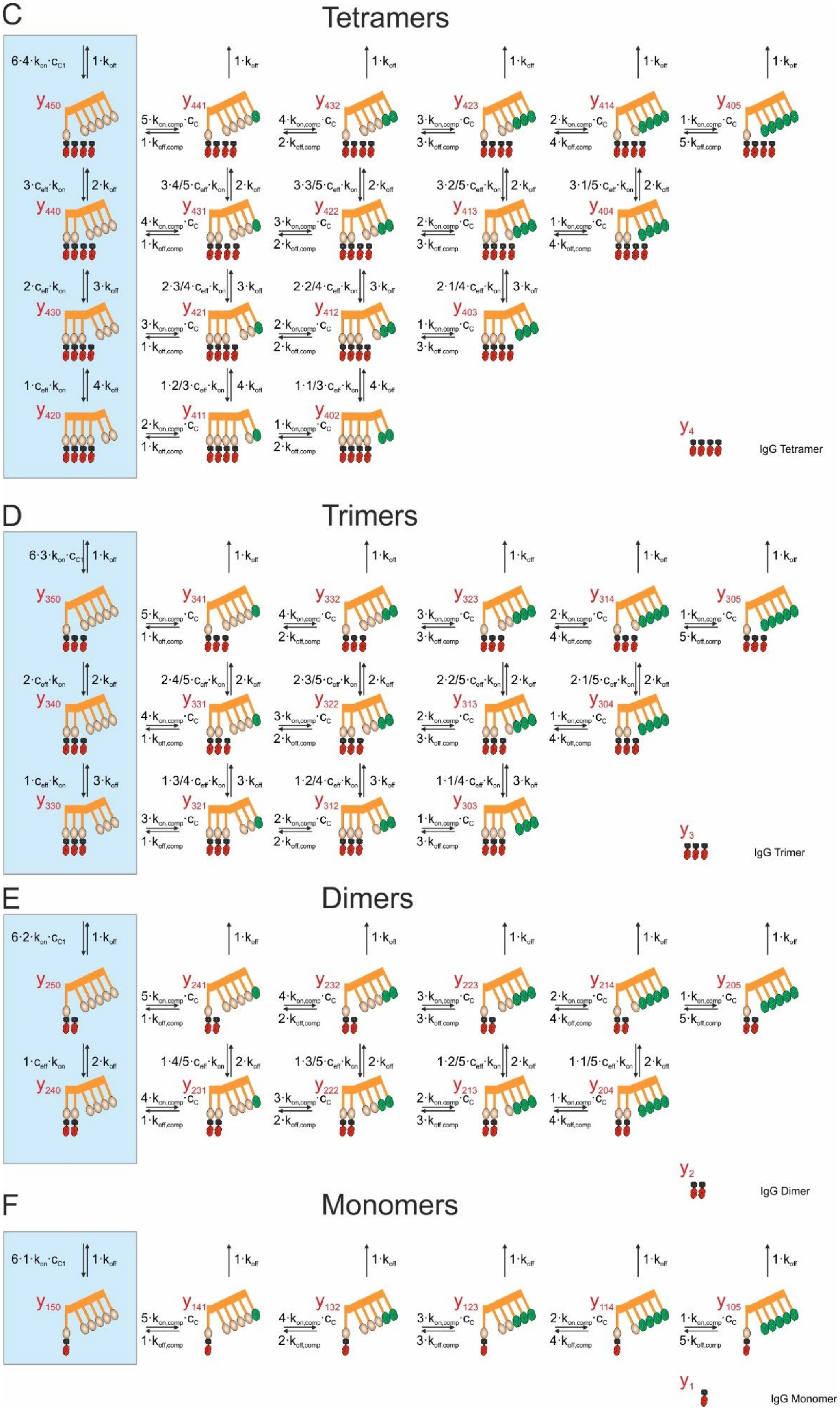
C
Tetramers
y450 y441 y432 y423 y414 y405
y440 y431 y422 y413 y404
y430 y421 y412 y403
y420 y411 y402
y4
IgG Tetramer
D
Trimers
y350 y341 y332 y323 y314 y305
y340 y331 y322 y313 y304
y330 y321 y312 y303
y3
IgG Trimer
E
Dimers
y250 y241 y232 y223 y214 y205
y240 y231 y222 y213 y204
y2
IgG Dimer
F
Monomers
y150 y141 y132 y123 y114 y105
y1
IgG Monomer

Figure S5. Mechanistic model of C1/C1q bound to IgG monomers-hexamers **(A-F)** in the absence (blue background) / presence of a competitor for gC1q. The interaction between the competitor and solution phase C1/C1q is not considered since the latter is already removed from solution when the competitor is introduced in our QCM experiments (Fig. 6).

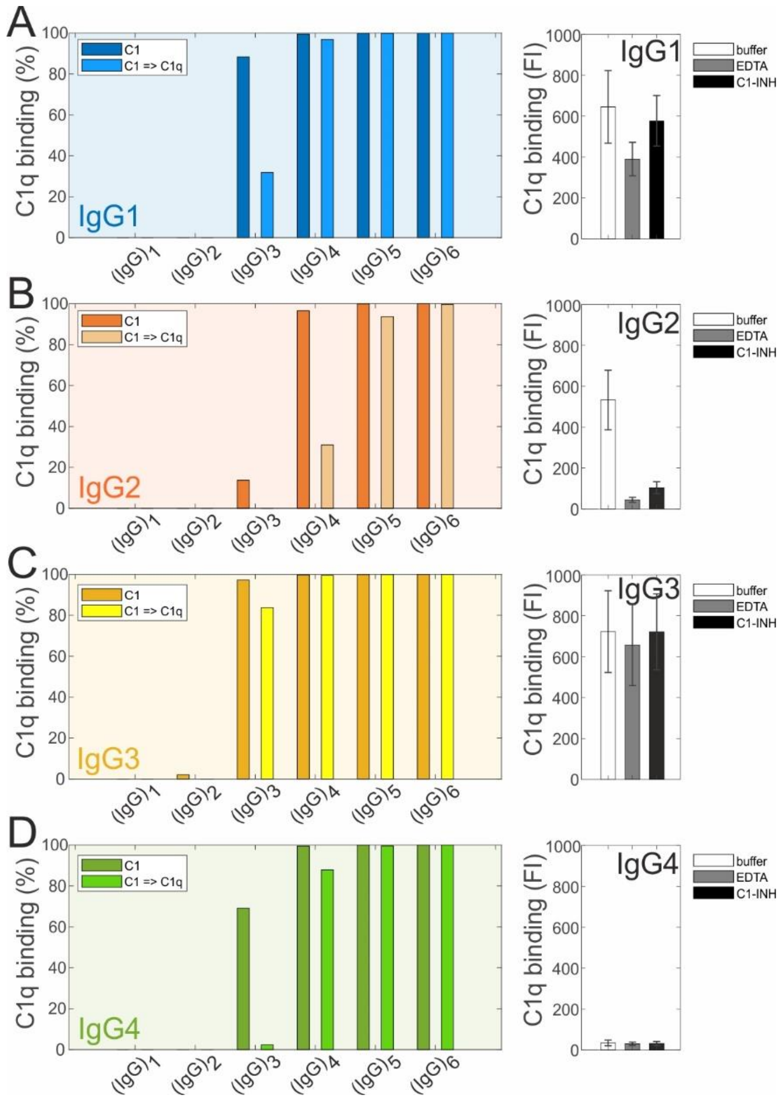

Figure S6. Model predictions for the removal of $C1r_2s_2$ from IgG-oligomer bound C1 by C1-INH or EDTA (right panels, data taken from (9)) on the stability of the remaining C1q – IgG oligomer complexes compared to the stability of the respective C1- IgG oligomer complexes. Following the experimental protocol given in (9), we simulated the binding of 3 nM C1 to IgG1-4 oligomers (incubation time: 30 min), after which the effective concentration c_{eff} (cf. Fig. 6B, Table 2) was either switched from 1.5 to 0.4 mM (light bars), or left at 1.5 mM (dark bars) mimicking the effect of $C1r_2s_2$ removal/retention. Bars represent the percentage of C1q molecules remaining bound to the respective IgG oligomers 60 min after the end of the incubation time, when in the experiment C1q binding was detected in a flow cytometer. At this point, both C1 and C1q are entirely dissociated from IgG1-4 monomers and dimers (some residual ~2 % C1 remained bound to IgG3 dimers), while removal of $C1r_2s_2$ from C1 affects C1q binding to higher IgG oligomers (> dimers) differently. **(A)** For IgG1, removal of $C1r_2s_2$ results in a reduction of C1q binding to IgG3 trimers from ~ 90 % to 30 %, while IgG1 tetramers to hexamers are mostly unaffected. The experimentally observed reduction in C1q binding (right panel) thus likely reflects the dissociation of C1q from IgG1 trimers. **(B)** In case of IgG2, only ~ 15 % of IgG2 trimers are predicted to be occupied by C1, which entirely dissociate when $C1r_2s_2$ is removed. C1q binding to IgG2 tetramers reduces to ~ 30 % upon removal of the proteases as compared to almost 100 % occupation by C1, while IgG2 pentamers and hexamers would both strongly bind C1 and C1q. This suggests that the strong reduction in C1q binding observed experimentally (right panel) is caused by the dissociation from IgG2 trimers and tetramers, while only a very small amount (if any) IgG2 pentamers and hexamers were present on

the antigenic surface. Consequently, the C3b deposition observed for IgG2 (which was comparable to IgG1 and IgG3 (9)) likely largely originates from C1 activation by IgG2 tetramers. **(C)** $C1r_2s_2$ removal leads to only minor dissociation of C1q from IgG3 trimers and does not affect binding to tetramers, pentamers and hexamers, which again correctly predicts the experimental observations (right panel). **(D)** While our model predicts strong C1 and C1q binding to hypothetical IgG4 tetramers-hexamers and a strong reduction of C1q binding for the removal of $C1r_2s_2$ from C1 bound to IgG4 trimers, neither C1/C1q binding (right panel) nor C3b deposition was experimentally observed for IgG4 (9), suggesting that IgG4 solely was present as monomers or dimers on the antigenic surface and was thus not detected, similar to what we have observed in our QCM experiments (Fig. 5).

Movie S1 (separate file). HS-AFM movie of two IgG3-DNP hexamers and four IgG3-DNP monomers bound to a DNP-SLB recorded at a scan speed of 2 s/frame. Scan size: 200 x 200 nm^2 (100 x 100 pixel). Color scale range: 0 - 15 nm.

Movie S2 (separate file). HS-AFM movie of an IgG3-DNP hexamer, an IgG3-DNP tetramer and IgG3-DNP monomers bound to a DNP-SLB recorded at a scan speed of 2 s/frame. Scan size: 200 x 200 nm^2 (100 x 100 pixel). Color scale range: 0 - 16 nm.

Movie S3 (separate file). HS-AFM movie of an IgG1-DNP hexamer and an IgG1-DNP monomer bound to a DNP-SLB recorded at a scan speed of 2 s/frame. Scan size: 200 x 200 nm^2 (100 x 100 pixel). Color scale range: 0 - 13 nm.

Movie S4 (separate file). HS-AFM movie of an IgG3-DNP hexamer bound to a DNP-SLB recorded at a scan speed of 2 s/frame. Scan size: 100 x 100 nm^2 (100 x 100 pixel). Color scale range: 0 - 15 nm.

SI References

1. R. N. de Jong, *et al.*, A Novel Platform for the Potentiation of Therapeutic Antibodies Based on Antigen-Dependent Formation of IgG Hexamers at the Cell Surface. *PLOS Biol.* **14**, e1002344 (2016).

2. J. L. Teeling, *et al.*, Characterization of new human CD20 monoclonal antibodies with potent cytolytic activity against non-Hodgkin lymphomas. *Blood* **104**, 1793–1800 (2004).

3. G. Hale, *et al.*, Removal of T cells from bone marrow for transplantation: a monoclonal antilymphocyte antibody that fixes human complement. *Blood* **62**, 873–882 (1983).

4. K. D. White, M. B. Frank, S. Foundling, F. J. Waxman, Effect of immunoglobulin variable region structure on C3b and C4b deposition. *Mol. Immunol.* **33**, 759–768 (1996).

5. D. R. Burton, *et al.*, Efficient neutralization of primary isolates of HIV-1 by a recombinant human monoclonal antibody. *Science* **266**, 1024–1027 (1994).

6. C. A. Diebolder, *et al.*, Complement Is Activated by IgG Hexamers Assembled at the Cell Surface. *Science* **343**, 1260–1263 (2014).

7. S. Yamamoto, *et al.*, Automated homogeneous liposome-based assay system for total complement activity. *Clinical Chemistry* **41**, 586–590 (1995).

8. D. Ugurlar, *et al.*, Structures of C1-IgG1 provide insights into how danger pattern recognition activates complement. *Science* **359**, 794–797 (2018).

9. S. A. Zwarthoff, *et al.*, C1q binding to surface-bound IgG is stabilized by C1r2s2 proteases. *PNAS* **118** (2021).
